# Anisotropic quasi-one-dimensional layered transition-metal trichalcogenides: synthesis, properties and applications†

**DOI:** 10.1039/d0ra07160a

**Published:** 2020-10-02

**Authors:** Abhinandan Patra, Chandra Sekhar Rout

**Affiliations:** Centre for Nano and Material Sciences, Jain University Jain Global Campus, Jakkasandra, Ramanagaram Bangalore-562112 India r.chandrasekhar@jainuniversity.ac.in csrout@gmail.com

## Abstract

The strong in-plane anisotropy and quasi-1D electronic structures of transition-metal trichalcogenides (MX_3_; M = group IV or V transition metal; X = S, Se, or Te) have pronounced influence on moulding the properties of MX_3_ materials. In particular, the infinite trigonal MX_6_ prismatic chains running parallel to the *b*-axis are responsible for the manifestation of anisotropy in these materials. Several marvellous properties, such as inherent electronic, optical, electrical, magnetic, superconductivity, and charge density wave (CDW) transport properties, make transition-metal trichalcogenides (TMTCs) stand out from other 2D materials in the fields of nanoscience and materials science. In addition, with the assistance of pressure, temperature, and tensile strain, these materials and their exceptional properties can be tuned to a superior extent. The robust anisotropy and incommensurable properties make the MX_3_ family fit for accomplishing quite a lot of compelling applications in the areas of field effect transistors (FETs), solar and fuel cells, lithium-ion batteries, thermoelectricity, *etc.* In this review article, a precise audit of the distinctive crystal structures, static and dynamic properties, efficacious synthesis schemes, and enthralling applications of quasi-1D MX_3_ materials is made.

## Abstract 2

The strong in-plane anisotropy and quasi-1D electronic structures of transition-metal trichalcogenides (MX_3_; M = group IV or V transition metal; X = S, Se, or Te) have pronounced influence on moulding the properties of MX_3_ materials.

## Graphical abstract

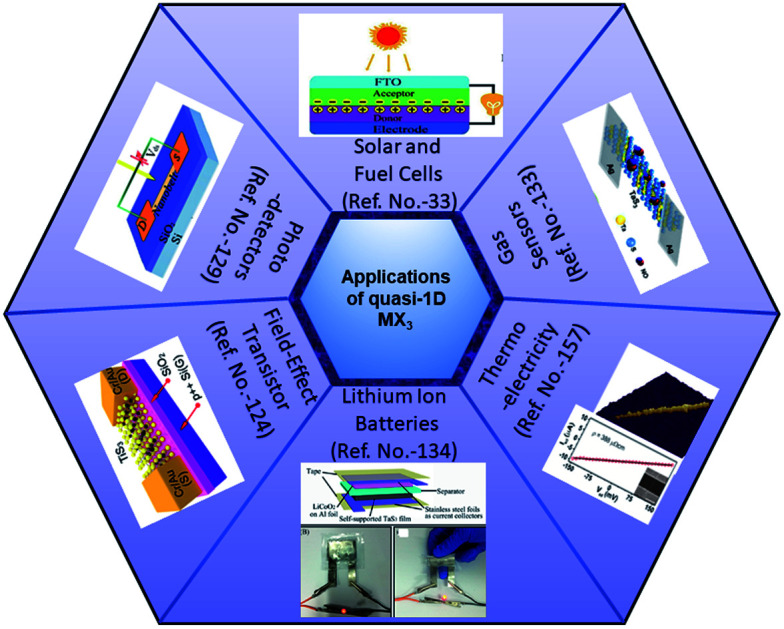

## 1. Introduction

The many pioneering studies based on the modern miracle material graphene^1^ paved the way for other monolayered and few-layered two-dimensional transition metal dichalcogenides (TMDCs) to be studied. They gained huge attention due to their strong tunable mechanical, electrical, optical, and physiochemical properties originating from the low dimensionality and quantum confinement effects. In accordance with the above excellent properties, they are useful for various optoelectronic and energy storage applications. Another noteworthy family of two-dimensional materials is transition metal trichalcogenides (MX_3_), which have been a source of attraction for a couple of years. These MX_3_ materials possess typical electrical, optical, magnetic, and physical properties that are quite anisotropic. Therefore, linking the benefits of 2D materials (elasticity, robustness, ease of synthesis, and huge surface to volume ratios) with excellent quasi-one-dimensional (1D) properties can be reasonably fruitful for application in the unexplored directions of nanoelectronics and nanotechnology.

The weak van der Waals amalgamated crystal structures of MX_3_ architecturally and chemically constitute a very distinct family of compounds, where M (group IV, V, or VI) represents a metal atom and X is a chalcogen atom (S, Se, or Te), and the M–X bond is ionic–covalent in nature.^6–9^ The compact in-plane anisotropy and quasi one-dimensional properties of monolayer and few-layer MX_3_ contribute a huge amount to configuring every single property of these materials. The prismatic MX_6_ chains of MX_3_ are extended along the *b*-axis to provide strong anisotropy. Pressure, temperature, and tensile strain can tune the electronic properties, and charge density wave transportation plays a pivotal role in making metal trichalcogenides a prime material of interest in the nanoscience and materials science communities. Instead of bulk or layered MX_3_, monolayer metal trichalcogenides are of considerable interest in both experimental and theoretical investigations. Monolayer TiS_3_ and TaS_3_, fabricated through mechanical exfoliation, show amazing superconductivity and charge density wave phenomena. This breakthrough accounts for their pronounced, unparalleled, and plentiful applications in the field of nanoelectronics.

Wide amounts of research have been ongoing concerning the flexible fabrication of MX_3_ materials, and attempts to shed several layers to obtain monolayers have also been made in the past few years through many emerging top-down synthesis methods. These new-fangled methods include chemical vapour deposition (CVD), chemical vapour transport (CVT), solvothermal methods, mechanical and chemical exfoliation, *etc.* As far as this review is concerned, we report cumulative information regarding crystal structures, elementary properties, synthesis procedures, applications, and experimental and theoretical developments related to MX_3_-based materials. Firstly, emblematic crystal structures and typical electrical, optical, magnetic, and CDW transport property characteristics are thoroughly discussed. Secondly, the fabrication of these materials is looked into. Lastly, the wide variety of applications of MX_3_, including field emission transistors,^124–126,142^ solar and fuel cells,^139,143–148^ photo-detectors and -sensors,^33,139,150,151^ lithium ion batteries,^134,152–155^ and thermoelectricity,^156–161^ are discussed with proper reference to previous studies.

## 2. Classification and crystal structures of MX_3_

Layered MX_3_ materials have emerged as a modern development with interesting electrical and optical properties due to their in-plane anisotropy.^2–4^ With robust structural in-plane anisotropy, MX_3_ materials combine the boons of both 2D layered material properties and quasi-1D material properties. Materials in the MX_3_ family are fascinating candidates for this purpose due to their reduced in-plane bonding symmetry.^2–4^ These MX_3_ materials can be classified into three categories (IV–X, V–X, VI–X) based on the metal (M) position in the periodic table. Here, X is the chalcogen element (S, Se, or Te); group IV elements are Ti, Zr, Hf, and Rf; group V elements are V, Nb, Ta, and Db; and group VI elements are Cr, Mo, W, and Sg. Among these materials, VI–X materials are reported to be amorphous^5^ and are less reported in the literature. A brief overview of the classification, crystal structures, and properties of widely studied MX_3_ materials (IV–X, V–X) is provided in Table 1.

**Table 1:** An overview of the crystal structures and properties of MX_3_

Group	Transition metal	S	Se	Te
IV	Ti	Monoclinic	No report	No report
		*a* = 4.973 Å, *b* = 3.433 Å, *c* = 8.714 Å; *β* = 97.74°		
		Diamagnetic; n-type semiconductor; band gap: 0.8–1 eV; Hall mobility: 30 cm^2^ V^−1^ s^−1^ (ref. 2 and 12)		
	Zr	Monoclinic	Monoclinic	Monoclinic
		*a* = 5.06 Å, *b* = 3.6 Å, *c* = 8.95 Å; *β* = 98.4°	*a* = 5.411 Å, *b* = 3.749 Å, *c* = 9.44 Å; *β* = 97.45°	*a* = 5.863 Å, *b* = 3.923 Å, *c* = 10.089 Å; *β* = 97.74°
		Diamagnetic; p-type semiconductor; band gap: 1.8–2.5 eV; Hall mobility: 26 cm^2^ V^−1^ s^−1^ (ref. 12 and 52)	Diamagnetic; n-type semiconductor; band gap: 1.1 eV; Hall mobility: 3.9 cm^2^ V^−1^ s^−1^ (ref. 29 and 164)	Semi-metallic; *T*_CDW_ = 63 K; *T*_c_ (superconductor) = 2 K (ref. 13 and 165)
	Hf	Monoclinic	Monoclinic	Monoclinic
		*a* = 5.08 Å, *b* = 3.58 Å, *c* = 8.96 Å; *β* = 98.4°	*a* = 5.31 Å, *b* = 3.73 Å, *c* = 9.525 Å; *β* = 97.18°	*a* = 5.879 Å, *b* = 3.902 Å, *c* = 10.056 Å; *β* = 97.98°
		Diamagnetic; p-type semiconductor; band gap: 1.9–3.1 eV; Hall mobility: 26 cm^2^ V^−1^ s^−1^ (ref. 52 and 165)	Diamagnetic; p-type semiconductor; band gap: 1.0 eV (ref. 3 and 7)	Semi-metallic; *T*_CDW_ = 93 K; *T*_c_ (superconductor) = 4.3 K (ref. 151 and 166)
V	Nb	Triclinic	Monoclinic	No report
		*a* = 4.963 Å, *b* = 6.730 Å, *c* = 9.144 Å; *β* = 97.17°	*a* = 10.009 Å, *b* = 3.48 Å, *c* = 15.629 Å; *β* = 109.47°	
		Diamagnetic; semiconductor; band gap: 0.8–1.1 eV (ref. 55 and 83)	Metal; *T*_CDW_ = 145 K and 59 K (ref. 75, 79, 94 and 164)	
		Monoclinic		
		*a* = 9.68 Å, *b* = 3.37 Å, *c* = 14.83 Å; *β* = 109.9°		
		Diamagnetic; semiconductor; band gap: 0.3–1.0 eV; Hall mobility: 10–2400 cm^2^ V^−1^ s^−1^ (ref. 12 and 13)		
	Ta	Orthorhombic	Monoclinic	No report
		*a* = 36.804 Å, *b* = 15.173 Å, *c* = 3.34 Å	*a* = 10.402 Å, *b* = 3.495 Å, *c* = 9.829 Å; *β* = 106.26°	
		Metal^8^	Metal; superconductor; *T*_c_ = 2.1 K (ref. 55, 79 and 164)	
		Monoclinic		
		*a* = 9.515 Å, *b* = 3.3412 Å, *c* = 14.912 Å; *β* = 109.99°		
		Metal^166^		

**Fig. 1:**
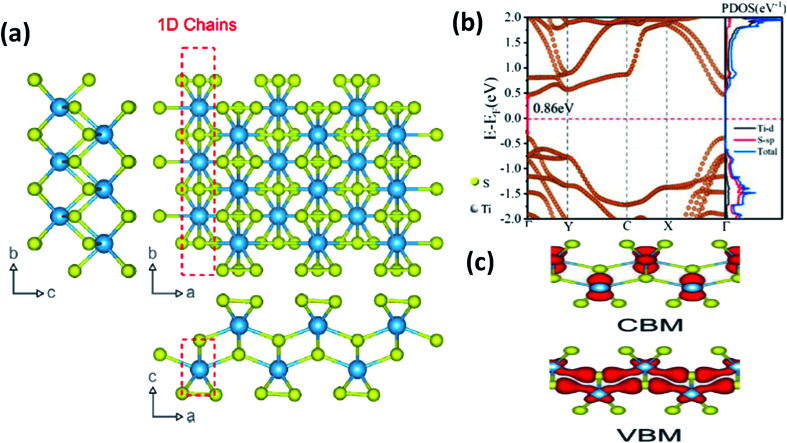
(a) The crystal structure of anisotropic TiS_3_ indicating the bond lengths between titanium and sulfur along the *b*-axis and *a*-axis, with shorter bonds along the *b*-axis; reproduced with permission from ref. 6, copyright: 2015, Springer Nature. (b) The calculated electronic energy band structure along the symmetry directions of the Brillouin zone (*Γ*–*Y*–*C*–*X*–*Γ*) and the projected density of states (PDOS) of the optimized structure of ML TiS_3_. The direct band gap *E*_g_ of ML TiS_3_ is indicated by the red arrows. The Fermi level is set to zero energy (the dashed line); reprinted with permission from ref. 8, copyright: 2019, American Chemical Society. (c) The charge distributions of the VBM and CBM states of monolayer TiS_3_; republished with permission from ref. 9, permission conveyed through Copyright Clearance Center, Inc.

In most transition metal trichalcogenides, MX_6_ trigonal prisms (M as the central atom and X dwelling in the prism triangular base) are stacked so as to obtain MX_3_ trigonal prismatic chains, and these chains (aligned parallel to the *b*-axis of the unit cell) are covalently bonded through van der Waals forces [Fig. 1a].^6^ Due to this, these chalcogenides have the tendency to show 1D characteristics along with anisotropy. Depending on the X–X bond lengths in these MX_3_ crystals, three types of chain arrangement are possible,^2–4^ which are outlined below:

➢ A one-type chain arrangement is present in ZrSe_3_, with Se–Se bond lengths of 0.234 nm and (Se_2_)^2−^ pairs forming in the trigonal base.

➢ A two-type chain arrangement is present in TaSe_3_, with Se–Se bond lengths of 0.258 nm and 0.291 nm (exceeding the value of ZrSe_3_). TiS_3_ crystals are an example of this type, with the arrangement: Ti^4+^S^2−^(S_2_)^2−^.

➢ A three-type chain arrangement is present in NbSe_3_ unit cells, with three Se–Se bonds at the base of the trigonal prismatic chains, short (III), mean (I), and long (II), having bond lengths of 0.237 nm, 0.248 nm, and 0.291 nm, respectively. The short bond length (0.237 nm) is ascribed to (Se_2_)^2−^, which supports the weakening of bonds between two Se atoms and strengthens the bonds between Nb and Se atoms. These types of arrangement create an intermediate situation with Nb^4+^ and Nb^5+^.

### 2.1. Crystal structures and properties of IV–X type MX_3_

As shown in Table 1, TiX_3_, ZrX_3_, and HfX_3_ (X = S, Se, or Te) are in the group-IV–X family, falling into the *P*2_1_/*m* space group, and have been investigated for their remarkable physical and chemical properties. Of these, the crystal and band structures of TiS_3_ are shown in Fig. 1a–c.^6,8,9^ Different Ti–S bond lengths along the *a* (2.65 Å) and *b* (2.45 Å) axes consequently result in highly conducting 1D chains parallel to the *b*-axis and turn out to be the reason for the solid anisotropic properties.^2,7^ In Fig. 1b,^8^ the red dashed lines show the Ti one-dimensional chain, which is also covalently bonded laterally with the *a*-axis, materializing sheets and networks through van der Waals forces. The unit cell of monolayer TiS_3_ is supposed to be a rectangle, whereas for bulk TiS_3_, it is a monoclinic crystal with the measured lattice constants of *a* = 4.973 Å, *b* = 3.433 Å, *c* = 8.714 Å, and *β* = 97.74°.^2,6^ The nuclear binding energy of bulk TiS_3_ is ∼0.22 per unit cell.^6,10,11^ Significantly, there are no reports on TiSe_3_ and TiTe_3_.

All members of the ZrX_3_ and HfX_3_ family take the form of monoclinic crystals, whose lattice parameters are summarized in Table 1. As discussed, the layered-type crystal structure of MX_3_ has the metal ion (M) in the centre of a slanted trigonal prism; stacked trigonal faces form separated columns that arrange along the *b*-axis, and the base triangle has a shape with two sides much longer than the third one.^12–15^ This criterion is satisfied by the formulation (X^2−^)(X_2_^2−^), with the metal oxidation state being M^4+^ (d^0^), leading to empty d-block bands. Due to this fact, most MX_3_ compounds are reported to be semiconducting, with a band gap of ∼1–2 eV, except ZrTe_3_ and HfTe_3_, which are semi-metallic with superconducting properties at low temperatures.^12–15^ The crystal structure of ZrTe_3_ is presented in Fig. 4a and b.^15^

#### 2.1.1. Optical, electronic, and anisotropic properties of IV–X-type MX_3_

TiS_3_ is found to be an n-type semiconductor with an energy band gap of 0.8–1.0 eV, and for *N* = 5 layers, the band gap is just 24 meV less than that of a single layer (*N* = 1). For that reason, having robust conduction band maximum (CBM) and valance band maximum (VBM) energy states, the energy gap of TiS_3_ does not change depending on the layer thickness.^11^ First principles calculations on the electronic structures of mono- and few-layered films demonstrate that the properties are quite fixed and do not depend on the number of layers, vertical strain, and piling order.^9^ Gomez *et al.* measured the electronic band gap and optical band gap to be 1.20 eV (with a rectification factor of 0.08 eV) and 1.07 eV (with a rectification factor of 0.01 eV), respectively, hence demonstrating the exciton energy (the difference between the electronic band gap and optical band gap) to be 130 meV.^16^ Biele *et al.* demonstrated that tuning from a direct to an indirect band gap in TiS_3_ is possible *via* the application of compressive strain during the course of normal electrical transport. *Ab initio* calculations and optical absorption experiments confirmed a band gap increase of 9% (from 0.99 to 1.08 eV) upon tensile stress stimulation.^17^ DFT calculation reports on the strain engineering of TiS_3_ monolayers demonstrate that the degree of anisotropy in mobility and effective mass can be reformed using tensile strain.^11^

**Fig. 2:**
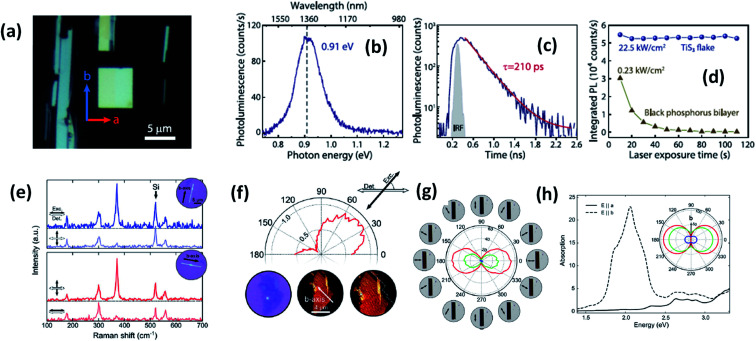
The optical properties of TiS_3_ flakes. (a) An optical microscope image showing the *a* and *b* axis directions and infrared light emission from TiS_3_. (b) Photoluminescence and (c) time-resolved photoluminescence spectra and (d) the photostability of a TiS_3_ flake compared to black phosphorus. Reproduced with permission from ref. 18, copyright: 2019, IOP Science. (e) Raman spectra of TiS_3_ ribbons with horizontal excitation and detection polarization. (f) The intensity of the 370 cm^−1^ Raman peak of a 3 nm-thick flake (3–4 layers) as a function of the excitation polarization angle (ref. 18). (g) The transmittance of the red, green, and blue channels as a function of the excitation polarization angle. (h) Calculated absorption spectra when the field is aligned parallel to the *b*-axis (dashed line) and the *a*-axis (solid line) with the inset showing the transmittance in the *a*–*b* plane for red (1.9 eV), green (2.4 eV), and blue (2.72 eV) excitation energies. Reproduced with permission from ref. 6; copyright: 2015, Springer Nature.

**Fig. 3:**
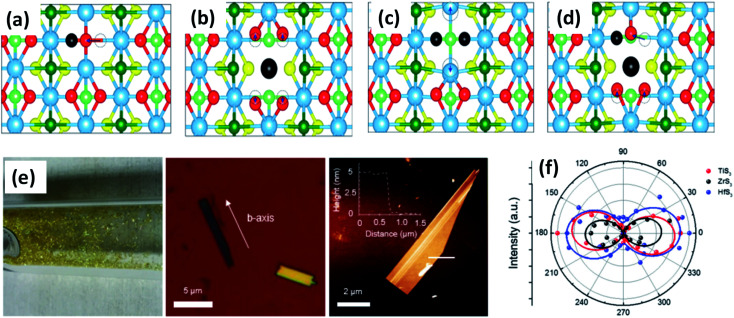
Top views of relaxed monolayer TiS_3_ with (a) S, (b) Ti, (c) double S, and (d) TiS vacancies. The black atoms illustrate removed atoms, and the dashed circles show the initial positions of the displaced atoms. Reproduced with permission from ref. 19, copyright: 2015, American Chemical Society. (e) An optical photograph of HfS_3_ needles grown on the inner walls of a quartz ampoule, an optical image of ZrS_3_ flakes exfoliated onto SiO_2_ substrates with the *b*-axis direction shown, and an AFM image of an exfoliated TiS_3_ flake with a thickness of ∼5 nm. (f) A polar plot of mode III at 372 (red), 320 (black), and 321 (blue) cm^−1^, which corresponds to thin TiS_3_, ZrS_3_, and HfS_3_ flakes, respectively. Republished with permission from ref. 24, permission conveyed through Copyright Clearance Center, Inc.

Experimental reports by Khatibi *et al.* showed that TiS_3_ emits near infrared (NIR) light centred at about 0.91 eV (1360 nm) with undeviated polarized anisotropic photoluminescence with a radiation life span of 210 ps (Fig. 2a–d).^18^ The dependence of emission on the excitation power and temperature demonstrated the dominant behaviour of free and bound electron–hole pairs over excitonic radiation at room and low temperature, correspondingly. TiS_3_ is reported to show excellent stable emission compared to other 2D materials, such as black phosphorus, MoTe_2_, *etc.*^18^ TiS_3_ is one of the appealing materials in the MX_3_ family due to its anisotropic optical properties. By using a force field along either the *a*-axis or *b*-axis and carrying out detection at intermediate angles between *a* and *b*, the anisotropic optical properties of 2D TiS_3_ are investigated.^6^ The anisotropic optical properties survey demonstrated strong emission anisotropy along the *b*-axis (25% higher emission intensity than along the *a*-axis). Island *et al.* inspected the strong in-plane anisotropy of TiS_3_ through angle-resolved polarization Raman spectroscopy (ARPRS).^6^ Compared to at room temperature, at 25 K, the in-plane conductivity in terms of anisotropy increases 2.09-fold. Fig. 2e and f^6^ shows Raman studies of TiS_3_ ribbons under different polarization conditions. Taking the *b*-axis as the favourable growth axis, four distinguishable Raman peaks (excluding one peak due to the silicon substrate) due to TiS_3_ were observed from an isolated nanoribbon on the SiO_2_/Si substrate. It is reported that the intensities of all the modes can be altered *via* changing the polarization angle, whereas the peak close to 370 cm^−1^ is found to be reliant on alignment between the polarization of the excitation laser and the *b*-axis. Furthermore, the anisotropy of the photosensitivity is verified based on the transmission of TiS_3_ through characteristic angles (Fig. 2h).^6^ The transmission reached a minimum value when the angle between the polarized excitation light source and the elongated side of the flakes was 180° (parallel to each other) in correspondence with the *b*-axis. The collected optical transmission data confirmed that TiS_3_ flakes exhibit robust linear dichroism, where the properties along the *b*-axis dominate those of the *a*-axis by as much as 30-fold, which is commendable for a 2D material.^6^ Compared to common semiconductors and other 2D TMDCs, TiS_3_ nanoribbons have a high exciton binding energy, which makes them an excellent applicant for use in optoelectronic devices and various related applications. Iyikanat *et al.* calculated the band gap of TiS_3_ using the PBE (Perdew–Burke–Ernzerhof) approximation and HSE06 (Heyd–Scuseria–Ernzerhof functional) correction to be 0.23 eV and 1.05 eV, respectively.^19^ The effects of S, Ti, TiS, and double S vacancies in TiS_3_ on its optical and electrical properties were also discussed based on DFT calculations by the above group (Fig. 3a–d).^19^ Of the four types of vacancies, except for S vacancies, the others lose their semiconducting properties and become metallic, resulting in a net magnetic moment. However, the low formation energy in the case of S vacancies assists in the opening of the energy gap of TiS_3_ monolayers. The calculated magnetic moments for the Ti, TiS, and double S vacancies of TiS_3_ are found to be 0.5, 0.8, and 0.3 *μ*_B_ per supercell, respectively.^19^ Kang *et al.* reported that the microelectronic properties of TiS_3_ nanoribbons strongly depend on the side, whether it is *a* or *b*.^20^*a*-TiS_3_ nanoribbons are reported to have the properties of a metal, having a width-dependent band gap, whereas *b*-TiS_3_ is a semiconductor with a direct energy gap, which can be regulated by strain, is nearly self-governing, and does not hinge on the ribbon width.^20^

ZrS_3_ is reported to be a p-type semiconductor, with resistivity of 15 Ω cm at room temperature, a direct optical energy gap of 1.8–2.5 eV, and two indirect optical energy gaps of 2.055 eV (when the field is along the *b*-axis) and 2.058 eV (when the field is normal to the *b*-axis) at 4.2 K.^4^ Angle-resolved photoemission and optical measurements of ZrX_3_ (X = S or Se) and HfSe_3_ predict that, due to spin–orbit interactions, there is splitting of the highest occupied band into two parallel bands. The split energy rises from S to Se in the above compound, and the absorption spectra are found to be highly anisotropic considering the phonon modes and electronic transitions.^21^ Schairer *et al.* evaluated the optical gap energies of ZrS_3_ and HfS_3_ to be 2.8 and 3.1 eV.^12^ In another report, Jandl *et al.* confirmed the presence of two polytypes in accordance with the recombination of phonon replicas of excitons localized in band tails.^22^ Pant *et al.* reported the indirect band gap of ZrS_3_ to be 1.88 eV, and angle-resolved photoluminescence (PL) spectroscopy studies of ZrS_3_ nanosheets demonstrated that they are highly anisotropic, which is manifested by the large PL intensity variation with polarization direction.^23^ Raman spectroscopy and angle-resolved studies are fast and non-destructive optical methods to probe the anisotropic nature of MX_3_. In this regard, Kong *et al.* collected Raman data from MX_3_ flakes (TiS_3_, ZrS_3_, and HfS_3_) with the longer edge (the anisotropic *b*-axis) aligned parallel to both the polarization direction of the laser and the polarization exposure path of the Raman spectrometer.^24^ To the contrary, for TiNbS_3_ alloy trichalcogenides, it is not possible to determine the anisotropic direction due to the loss of anisotropy and common defects present in the random distribution of quasi-1D MX_6_ chains.^24^ Wang *et al.* verified the anisotropic effects in ZrS_3_*via* angle-resolved absorption and angle-resolved Raman spectroscopy, and angle-resolved photocurrent studies, which gave a vibrant impression that all the properties were boosted along the *b*-axis. Angle-resolved spectroscopy studies revealed dichroic ratios of 1.73 and 1.14 for the ZrS_3_ nanoribbons when excited by different laser source wavelengths, *i.e.*, 450 nm and 532 nm, respectively, considering the photocurrent density.^25^ The change in dichroic ratio is owing to the variation of the offset angle from the *b*-axis. Jin *et al.* showed the widening and trivial shifting of peaks in the Raman spectra due to the phonon confinement effect in the cases of ZrS_3_ and HfS_3_ nanobelts.^26^ Likewise, TiS_3_ Raman spectra collected by Pawbake *et al.* showed the shifting of the peaks towards lower wavenumbers upon an increase in temperature (88 K to 570 K), which is attributed to thermal expansion of the lattice and anharmonic vibration.^27^ Apart from temperature, pressure-dependent Raman spectroscopy studies were done by Wu *et al.*, which describe the unorthodox negative pressure dependence of the A^S–S^_g_ S–S molecular mode in contrast to the expected stiffening of other peaks. Numerous modes of TiS_3_ are reported to be doubly degenerate at ambient pressure, whereas at high pressure, this effect vanishes.^28^

Patel *et al.* calculated the direct and indirect energy gaps of ZrSe_3_ to be around 1.47 eV and 1.1 eV, respectively. They also found that by means of an escalation in temperature in the vicinity of 303–403 K, the anisotropy and resistivity could be decreased and increased, respectively.^29^ In agreement with quasi particle self-energy correction, Zhou *et al.* found the indirect band gap of ZrSe_3_ to be 1.63 eV.^30^ Felser *et al.* established a dependent relationship between the density of states and the shape of the Fermi surface in Te–Te interprism interactions in ZrTe_3_.^14^ Zeng *et al.* showed that growing strain in HfS_3_ resulted in a switch from an indirect to a direct band gap of 2.2 eV, whereas Tao and his group calculated the indirect and direct optical energy gaps of HfS_3_ nanobelts to be 1.73 and 2.19 eV, respectively.^31,32^ As shown by photoluminescence (PL) studies, the nanobelts displayed strong emission at 483, 540, and 600 nm in response to excitation at 400 nm.^32^ Likewise, Zhao *et al.* calculated the band gaps for Zrs_3_, Zrse_3_, Hfs_3_, and Hfse_3_ to be 1.13, 0.23, 1.08, and 0.05 eV, respectively. According to their study, the p state of the chalcogen (governing the VBM) and the d state of the transition metal (governing the CBM) combined to form the band gap of the MX_3_ monolayers.^33^

**Fig. 4:**
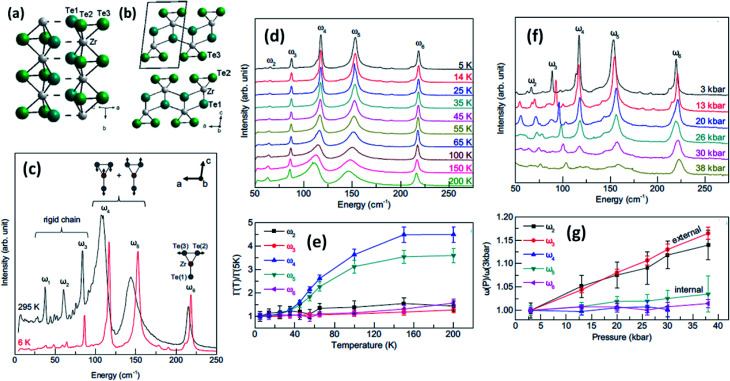
The crystal structure of ZrTe_3_: (a) quasi-one-dimensional trigonal prism packing along the *b* axis and (b) a quasi-two-dimensional ZrTe_3_ layer along the *a*–*c* plane; reprinted with permission from ref. 15, copyright: 2013, American Physical Society. (c) Raman spectra of ZrTe_3_ at 6 K and 295 K. (d and e) Temperature-dependent Raman spectra at ambient pressure and (f and g) pressure-dependent Raman spectra at *T* = 3 K. Reprinted with permission from ref. 36, copyright; 2013, American Physical Society.

Trisulphides, for example ZrS_3_- and HfS_3_-based structures, are reported to exhibit two dozen normal modes at the centre of the Brillouin zone and belong to the *C*_2h_ space group.^26,34,35^ Each mode is interconnected with a clutch of atomic vibrations represented by A_g_, B_g_, A_u_, and B_u_. Among these, the A_u_ and B_g_ vibrations are parallel to the MX_6_ trigonal prism chains, and the A_g_ and A_u_ bands are perpendicular to the chains. Gleason *et al.* investigated the pressure-induced phases of ZrTe_3_ and also analysed the temperature-dependent Raman spectra.^36^ It is reported that some specific phonon modes endure a dramatic linewidth reduction near the charge density wave temperature (*T*_CDW_), indicating the strong coupling of phonons with electronic degrees of freedom concomitant with the CDW (Fig. 4). Typical Raman spectra from a ZrTe_3_ crystal are shown in Fig. 4c at 295 K and 6 K with normal mode displacement patterns. The lower-energy modes (ω_1_–ω_3_) and higher-energy modes (ω_4_–ω_6_) are allocated primarily to vibrations of the trigonal prismatic rods, inwardly and outwardly, respectively. Changes in the widths of the Raman bands at different temperatures (Fig. 4d and e)^36^ are observed, with the large reductions in the widths of ω_4_ and ω_5_ demonstrating strong coupling between these bands. Similarly, the downfall of the long-range-order (LRO) of the rods is attributed to the suppression of phonon bands allied to internal vibrations of the ZrTe_3_ prismatic rods at pressures above 10 kbar (Fig. 4f and g).^36^

#### 2.1.2. Electrical and magnetic properties of IV–X-type MX_3_

The electronic superstructure and projected density of states (PDOS) along the symmetry direction of the Brillouin zone of the augmented assembly of monolayer TiS_3_ are shown in Fig. 1b.^8^Fig. 1c depicts the distribution of charge in the VBM and CBM of monolayer TiS_3_.^9^ These details give a clear-cut idea about the high carrier mobility of 2D TiS_3_ monolayers. The electron mobilities of TiS_3_ are reported to be high and anisotropic, with electron mobility of 13.87 × 10^3^ cm^2^ V^−1^ s^−1^ along the *b*-axis, which is about 14 times greater than that along the *a*-axis (1.01 × 10^3^ cm^2^ V^−1^ s^−1^), while the hole mobility along the *a*-axis is 1.21 × 10^3^ cm^2^ V^−1^ s^−1^, about eight times higher than that along the *b*-axis (0.15 × 10^3^ cm^2^ V^−1^ s^−1^).^10^ DFT calculation reports on the strain engineering of TiS_3_ monolayers demonstrated that the extent of the anisotropy of both mobility and effective mass changed as a result of the influence of tensile strain. Strain engineering leads to an order-of-magnitude increase in the mobility of acoustic phonons at 300 K (100 K), *i.e.*, from 1.71 × 10^4^ (5.13 × 10^4^) cm^2^ V^−1^ s^−1^ to 5.53 × 10^5^ (1.66 × 10^6^) cm^2^ V^−1^ s^−1^.^11^ Investigations into the temperature dependence of resistance along the chains (*b*-axis) and across the chains (*a*-axis) of TiS_3_ are reported to show non-linear conductivity. The observed non-linear conduction in TiS_3_ brings to mind quasi-1D conducting behaviour with a sliding charge density wave.^37^

In quasi-1D conductors, the condensations of electrons into CDWs arises due to Fermi surface instability, and they form a deformable medium that affects their overall static and dynamic properties, giving rise to metallic stability and hysteresis. Low-dimensional materials, including anisotropic 2D MX_3_ layered structures, from time to time lose their LRO and orthodox symmetry, resulting in Fermi surface instability and forming a presumed charge density wave (CDW).^38,39^ ZrTe_3_ is one attention-grabbing material in the type IV–X MX_3_ family, which is reported to have anisotropic nature, with a CDW at 63 K and superconductivity at 2 K.^14,15,40–42^ Other reports on ZrTe_3_ revealed a CDW transition at 63–70 K due to resistivity inconsistency along the *a*-axis, but not along the conventional anisotropic *b*-axis.^41,43^ Canadell *et al.* predicted that ZrTe_3_ is a type-B structure with shorter X–X contacts between adjacent MX_3_ units. The type-B structure of ZrTe_3_ plays a crucial role in determining the semi-metallic nature of the ZrTe_3_ chains.^13^ Zhu *et al.* reported a comparison of the electrical and superconducting properties of ZrTe_3_ single crystals prepared at low (735 °C) and high (950 °C) temperatures.^15^ From resistive, colorimetric, and magnetic studies, bulk superconductivity is perceived in the HT-ZrTe_3_ crystals with the help of doping after a certain temperature, *i.e.*, 4 K, but not in the LT-ZrTe_3_ crystals. The difference in electrical properties is attributed to the suppression of CDWs through growth-induced structural disorder at high temperature.^15^ Polarized Raman measurements and first principles calculations demonstrated that precise structural vibrational arrangements from longitudinal distortions of the Te(ii)–Te(iii) chains had a strong association with the conduction of electrons, leading to the formation of CDWs in ZrTe_3_.^44–46^ Pressure-dependent electrical properties investigations revealed that the CDW transition temperature (*T*_CDW_) of ZrTe_3_ was initially amplified, then diminished at 2 GPa, and quickly vanished at 5 GPa, but superconductivity was shown at a pressure level of up to 11 GPa.^47^ However, Hoesch *et al.* performed low-temperature and high-pressure single crystal X-ray diffraction studies along with *ab initio* DFT studies to show that the reported abrupt demise of the CDW phase is due to instability in the Fermi surface above 5 GPa.^48^ Zhu *et al.* reported the presence of different bands, including bands with flat and dispersive profiles, along with the amalgamation of chalcogen (high mobility) and metal (low mobility) derived bands in the Fermi surface of ZrTe_3_. Due to the suppression of long-range CDW order, superconductivity materialises in Se-doped ZrTe_3_.^49^ The superconductivity critical temperature (*T*_c_) increased up to 4 K, but after that, additional Se doping caused a reduction in *T*_c_ and filamentary superconductivity in ZrTe_3−*x*_Se_*x*_ (0 ≤ *x* ≤ 0.1).^49^ Polycrystalline ZrTe_3_ is testified to show a superconducting transition temperature of 5.2 K and a *T*_CDW_ value of ∼63 K, both co-existing at ambient pressure.^50^ The intercalation of Ag and Cu into ZrTe_3_ favoured an escalation in electrical conductivity and resulted in CDW anomalies, which is confirmed from DC magnetisation results, whereas it had no role on *T*_CDW_ and *T*_c_.^50^ Li *et al.* reported CDW formation at *T*_CDW_ = 93 K with typical anisotropy.^51^ Conductivity measurements parallel to the *a*-axis (*ρ*_*a*_) and *b*-axis (*ρ*_*b*_) of a HfTe_3_ crystal gave a direct indication of the disorder-related superconductor fluctuations in *ρ*_*b*_ at 4.3 K. Also, a superconducting phase with *T*_c_ = 1.7 K co-exists with a lower *T*_CDW_ value of 80 K in a polycrystalline sample, whose generation could be attributed to enriched disorder scattering or unintended carrier doping.^51^ A combined experimental and computational study by Hu *et al.* explored a new concept, which emphasizes that phonon–electron coupling helps in the construction of CDWs in ZrTe_3_. However, their study depicted that the breaching of fractional electronic gaps in the CDW state depends upon the phonon–electron momentum and coupling.^44^ The resistivity and Hall mobility at room temperature of ZrSe_3_ were calculated by Ikari *et al.* to be about 9 × 10^2^ Ω cm and 0.45 cm^2^ V^−1^ s^−1^, respectively.^52^

Lai *et al.* reported a comparative investigation into the magnetic properties and tensile strain response of *N-a*(*b*)-TiS_3_ nanoribbons, where *a*-TiS_3_ and *b*-TiS_3_ are nanoribbons reviewed along either the *a*- or *b*-axis and *N* indicates the number of Ti atoms in the monoclinic cell of the ribbons.^53^ It was found that the magnetic ground state was a ferromagnetic (FM) metal when *N* was equal to an odd number, whereas it behaved like an antiferromagnetic (AFM) metal when *N* was equal to an even number for *N-a*-TiS_3_ nanoribbons. Tensile strain (6%) could be used to tune 9-*a*(*b*)-TiS_3_ nanoribbons from a FM metal to a half metal. Similarly, tensile strain (4%) also could cause an AFM to FM transition in 10-*a*-TiS_3_ nanoribbons.^53^

### 2.2. Crystal structures and properties of V–X-type MX_3_

**Fig. 5:**
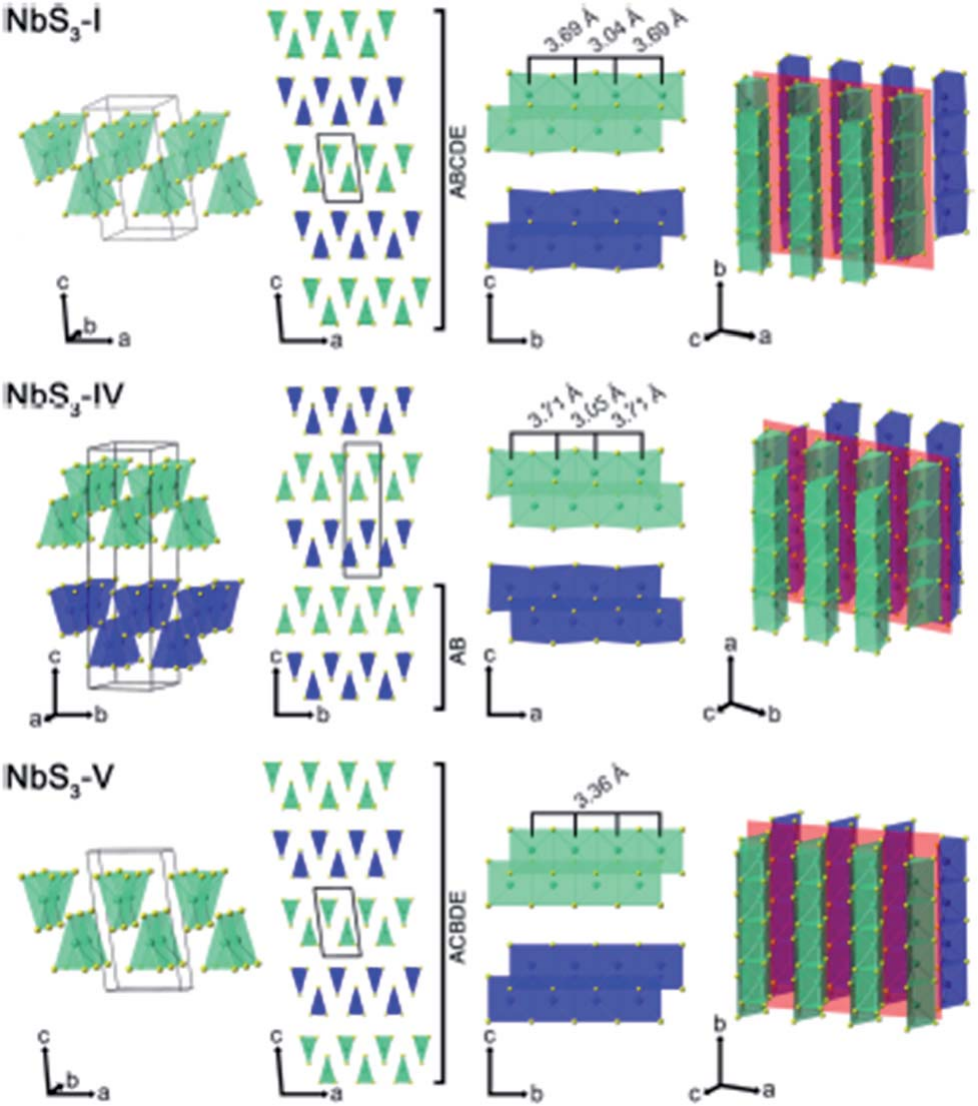
The crystal structure of NbS_3-I_, NbS_3-IV_, and NbS_3-V_: unit cells, chain cross-sections, layers, and perspective views. Reproduced with permission from ref. 55, copyright: 2017, Application Infrastructure Provider.

Apart from IV–X-type MX_3_, V–X-type MX_3_ has its own presence in the family of metal trichalcogenides with extraordinary physical, chemical, and electrical properties. NbX_3_ and TaX_3_ materials with X = S, Se, or Te belong to the V–X family and are strongly anisotropic materials consisting of conducting chains weakly attached by van der Waals forces (Table 1).^54,55^ Among these MX_3_ materials, NbS_3_ is reported to have six types of polymorph, which are summarized in Table 2. NbS_3-I_ has a monoclinic crystal structure, which was first proposed in 1960 and experimentally verified *via* single-crystal X-ray diffraction studies in 1978.^56,57^ NbS_3-I_ is reported to be a semiconducting material with a band gap of ∼0.66–1.0 eV, and its important feature is the bond-pairing between two Nb atoms along the NbS_3_ primary chain axis, with a bond length of ∼3 Å and creating a 3.7 Å space.^58–62^ The NbS_3-II_ polymorph was first acknowledged in 1978 following electron diffraction experiments, with weak pair satellite diffraction streaks that resolve into rows of spots upon modifications in temperature.^63,64^ It is proposed that NbS_3-II_ is an elevated structure of NbS_3-I_ with different types of chain accretion, and the rows of spots arise at random positions with analogous separation to NbS_3-I_ (Table 2). NbS_3-II_ is testified to have three CDWs at 150 K, 330–370 K, and 620–650 K.^61^ In 1982, Kikkawa and co-workers obtained monoclinic NbS_3-HP_*via* the extraordinary high-pressure modification of NbS_3_ synthesized at 700 °C with 2 GPa pressure.^65,66^ Zettl *et al.* produced NbS_3-III_ in 1982, which was distinctively different from the I and II phases.^67^ In NbS_3-III_, (001) reflections from the XRD data showed a similar *c*-axis as that reported for NbS_3-I_, but the monoclinic angle was increased to 98°–99° and *T*_CDW_ was ∼155 K. Zybtsev confirmed the low and high ohmic nature of NbS_3_, with CDW transitions at 150 K (both low and high ohmic) and 360 K (low ohmic).^66^ The low ohmic and high ohmic NbS_3_ was assigned as NbS_3-II_ and NbS_3-III_, respectively, with NbS_3-III_ designated as a sub-phase of NbS_3-II_. For the first time, Bloodgood *et al.* reported NbS_3-IV_ and NbS_3-V_ polymorphs of the NbS_3_ monoclinic crystal structure.^55^ The NbS_3-IV_ structure is constructed from NbS_6_ trigonal prismatic chains with a bond length between Nb atoms of 3.0448 Å, and the chain axis is along the *a*-axis instead of the *b*-axis like other conventional MX_3_ materials. For NbS_3-V_, the Nb–Nb bond length is 3.358 Å. The bonds in NbS_3-IV_ and NbS_3-V_ are along the chains, with an AB and ABCDE recapping categorization of chain bilayers, analogous to NbS_3-I_. The crystal structures of NbS_3-I_, NbS_3-IV_, and NbS_3-V_ are given in Fig. 5, with a complete overview of unit cells and layers from different perspectives. In the case of NbS_3-IV_, there are up to twice as many chains per unit cell compared to NbS_3-I_, since the *c*-axis is doubled, and it displays the properties of a semiconductor.^55^

**Table 2:** Summarized data of the crystal structures, synthesis conditions, and Nb–Nb bond lengths of different phases of NbS_3_ crystals

Material	Crystal structure *a*, *b*, c (Å); *α*, *β*, *γ* (°)	Synthesis conditions	Nb–Nb (Å)	Ref. no.
NbS_3-I_	4.963, 6.730, 9.144; 90, 97.17, 90	NbS_2_Cl_2_; 588 °C (source)/569 °C (sink); 48 h; slow cooling	3.045, 3.702	57
NbS_3-HP_	9.68, 3.37, 14.83; 90, 109.9, 90	Nb + S; 700 °C at 2 GPa; 0.5 h	3.370	131
NbS_3-II_	9.9, 3.4, 18.3; 90, 97, 90	Nb + S; 600 °C (source)/580 °C; 15 days	—	61 and 99
NbS_3-II_	9.1–9.6, 18.7–19.9, 3.4; 90, 97–98, 90	Nb + S; 500 °C	—	60
NbS_3-III_	∼5, —, ∼9; 90, 98–99, 90	Nb_2_ + S; 550 °C; 21 days; 400 °C, 48 h (48 h, air quenching)	—	67
NbS_3-IV_	6.7515(5), 4.9736(4), 18.1315(13); 90, 90.116(2), 90	Nb + S, I_2_ transport, 70 °C (source)/570 °C (sink), 10 days	3.0448(8), 3.7087(8)	55
NbS_3-V_	4.950(5), 3.358(4), 9.079(10); 90, 97.35(2), 90	Nb + S, 10% S, I_2_ transport, 670 °C (source)/570 °C (sink), 10 days	3.358(4)	55

In 1975, single-crystal NbSe_3_ was prepared by Meerschaut *et al.*, and single-crystal XRD studies established the monoclinic structure, consisting of inestimable selenium trigonal prismatic chains loaded on top of each other with shared triangular faces.^68–70^ Similar to other MX_3_-based 2D materials, the structural arrangement with strong chemical bonding anisotropy makes NbSe_3_ crystals a tempting material with novel physical and chemical properties. The Se–Se bond lengths are testified to be 2.37 Å and 2.49 Å, respectively, with CDWs at 145 K and 59 K, and the Nb–Se bonds are strongly covalent–ionic in nature.^70–73^ From scanning tunnelling microscopy studies of NbSe_3_, it is clear that there are three distinguishable chains (I, II, and III) in the NbSe_3_ unit cell at all temperature.^74^ The III chains are identified by their association with the CDW modulation vector *q*^1^, and the two remaining chains are named I and II.

**Fig. 6:**
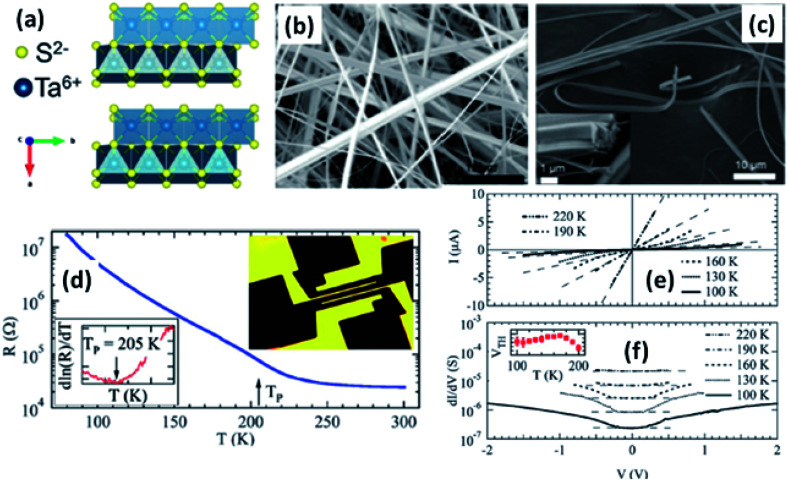
(a) The orthorhombic structure of TaS_3_. (b and c) FESEM images of TaS_3_ nanobelts. (d) Temperature-dependent resistance changes of a single nanoribbon, with the inset showing a fabricated device. (e) Current–voltage curves and (f) differential conductance as a function of voltage measured at specific temperatures, with the inset of (f) showing the temperature dependence of the threshold voltage. Republished with permission from ref. 104, permission conveyed through Copyright Clearance Center, Inc.

Among TaX_3_-based TMTCs, TaS_3_ and TaSe_3_ have been investigated due to their various fundamental microelectronic properties, which range from insulating to metallic conducting.^65,75^ TaS_3_ was first described by Blitz and Kocher in 1938 and later through XRD studies implemented by Jellinek in 1962.^76,77^ There are two commonly referenced structures of TaS_3_, a monoclinic state (m-TaS_3_) and an orthorhombic state (o-TaS_3_) (structure shown in Fig. 6a)^104^ (Table 1). Meerschaut *et al.* reported the complete structure of m-TaS_3_ with the space group *P*2_1_/*m*, and the lattice constants are *a* = 9.515(2) Å, *b* = 3.3412(4) Å, *c* = 14.912(2) Å, and *β* = 109.99°.^75^ o-TaS_3_ forms in the space group *C*222_1_ and the lattice constants are *a* = 36.804 Å, *b* = 15.173 Å, and *c* = 3.340 Å.^65,77^ A high-pressure m_hp_-TaS_3_ phase with the identical space group to m-TaS_3_ has similar lattice parameters, expect there is a deviation of *β* by 3°.^65^ m-TaS_3_ is a low-dimensional conductor with inestimable chains of tantalum atoms along the *b*-axis of the primitive cell. The remarkable properties of bulk and few-layer TaS_3_ are directly correlated to the crystal structure, specifically the chains of tantalum atoms and the *b*-direction of the unit cell, which leads to quasi-1D properties. TaSe_3_ belongs to the V–X group of MX_3_ with a monoclinic crystal structure, in which the unit cell consists of stacks of Ta atoms, each of which are fused to three Se atoms above and below the *b*-axis.^78,136^ The monoclinic crystal structure of TaSe_3_ was first recorded by Bjerkelund and Kjekshus in 1965 with *a* = 10.402 Å, *b* = 3.495 Å, *c* = 9.829 Å, and *β* = 106.26°.^79,80^ The inter-planar distance (nearly 4 Å) is more than the distance between the Ta atoms (3.495 Å), which gives clear evidence for the quasi-1D properties of TaSe_3_.^79^

#### 2.2.1. Electrical, optical, and magnetic properties of V–X-type MX_3_

As discussed, from structural and supporting spectroscopic data analysis, the V–X type MX_3_ phases can be represented by the ionic formula M^4+^(X_2_)^2−^X^2−^. The dichalcogenide groups, (X_2_)^2−^, are considered to be electron reservoirs and provide the MX_3_ phases with some astonishing properties. NbS_3-I_ is detailed to be a semiconductor with Nb–Nb (d^1^–d^1^) pairing in each chain. The pressure-dependent electrical properties of a quasi-one-dimensional NbS_3_ conductor demonstrate that whenever there is an enlargement in conductivity by six orders of magnitude at pressures of 3–4 GPa, there will be an insulator–metal transition.^81^ Furthermore, if the local conduction activation energy increases, then there will be an additional transition in harmony with the temperature dependence of resistance. Fedorov *et al.* reported the physical process and electrical properties of NbX_3_ thin-film FETs based on colloidal powder samples subject to ultrasonication in different solvents.^82^ In isopropyl-alcohol and ethanol–water mixtures, the concentrations of NbSe_3_ were found to be 0.332 g L^−1^ and 0.443 g L^−1^, respectively, which are the uppermost among all the used solvents. The highest carrier mobility is observed in NbS_3_ films obtained from the ethanol–water mixture colloidal solution [1200–2400 cm^2^ V^−1^ s^−1^], with n-type conductivity. The measured carrier mobility in NbS_3_–CH_3_CN colloidal solution is ∼10 cm^2^ V^−1^ s^−1^, with p-type doping.^82^ However, from quantum chemical studies, an understanding of electronic transitions (electron transfer from the molecular orbitals of bonding Nb–Nb bonds to anti-bonding Nb–Nb bonds) through the excitation of Nb–Nb bonds has allowed this to come to light as a novel concept.^83^ Wu *et al.* incorporated a low concentration of Ti (0.05–0.18%) into the NbS_3-I_ host matrix, which alters the phase from triclinic to monoclinic.^84^ Speculative studies suggest that the phase transition can be attributed to an increase in the entire energy of the triclinic phase *via* p-type doping induced by titanium atoms, which have one less electron in the valance shell compared to niobium. The alloyed NbS_3_ preserved its crystallinity, and optical and angle-resolved Raman measurements provided information about the crystalline anisotropy and its levels.^84^ From polarized infrared reflection and transmission studies, the absorption co-efficient for NbS_3-I_ was calculated by Itkis at two different temperatures (300 and 8.5 K).^60^ Electron–hole excitations across the Peierls gap^85^ and soliton-like excitations in the superstructure led to absorption in the spectral range >6700 cm^−1^ and <6700 cm^−1^, respectively.^60^ The UV-visible absorption spectrum (250–1000 nm) at 300 K and infrared and Raman spectra at 300 K (650–10 cm^−1^) and 100 K (650–180 cm^−1^) of NbS_3_ were obtained by Sourisseau *et al.*^86^ Their findings reveal the presence of absorbing semiconducting characteristics from UV spectroscopy, and they found 23 Raman bands and 18 infrared bands, which are close to the theoretical results.^86^

Monoclinic NbSe_3_ consists of three equidistantly placed metal chains with different (Se_2_)^2−^ groups, and it is reported to show metallic conductivity with two charge density wave temperatures of 145 K and 59 K.^69,87,88^ Chaussy *et al.* reported two phase-transition temperatures of NbSe_3_ crystals at 145 K and 59 K based on electrical resistivity, magnetic susceptibility, and heat capacity measurements.^87^ As per their revisions, when the temperature decreased, the resistivity (*ρ*) decreased, showing the behaviour of a metal, and saturation was reached below 10 K. The maximum specific heat capacity was attained at 49 K and gradually decreased at *T* = 0 K. It was found to be diamagnetic at 4.2 K, and for fibres and powder samples of NbSe_3_, the magnetization values were equal to 1.35 × 10^−7^ emu g^−1^ (attributed to a parallel orientation to the magnetic field) and 5 × 10^−7^ emu g^−1^ (attributed to a random orientation to the magnetic field), respectively.^87^ Whiskers of NbSe_3_ and Fe-doped NbSe_3_ nanowires also showed two anomalies in resistivity that were adjunct to the CDW transitions at 140 K and 50 K.^89^ The successful doping of Fe atoms can be observed based on the strengthening of both the threshold fields, *E*_T_1__ and *E*_T_2__. Four-probe resistivity measurements of single-crystal NbSe_3_ nanowires showed the expected CDW transitions at *T*_1_ = 142 K and *T*_2_ = 58 K, and there was no magnetoresistance above the higher *T*_CDW_ value and positive magnetoresistance below the lower *T*_CDW_ value.^72^ Ido *et al.* explored the effects of pressure on CDW formation and superconductivity in NbSe_3_*via* resistivity and diamagnetic measurements.^90^ It was observed that both the CDW transition temperatures *T*_1_ and *T*_2_ decreased steadily with an increase in pressure, while they later changed abruptly above 6 kbar pressure and tended to zero above *P*_c_ = 7.5 kbar (superconductivity appeared). The change in superconductivity arose from a clampdown on electron–phonon coupling.^90^ Latyshev *et al.* showed that the induction of oscillations into non-linear CDW conductivity could be attributed to columnar defects in NbSe_3_, which fluctuated with a magnetic field when the field was oriented parallel to the axes of the defects.^73,91^ Due to the CDW transitions, there were ample signs of electron–phonon scattering affecting the transport properties relating to the thermal conductivity of the lattice of NbSe_3_ nanowires.^92^ Ong *et al.* performed Hall measurements of NbSe_3_ and showed that at both *T*_CDW_ values there is an increase in Hall resistivity (*R*_H_).^93^ But this was self-regulating below 3 K, and with an increase in the field value, it saturated at 2.3 × 10^−6^ m^3^ C^−1^. Surprisingly, the value of *R*_H_ was 4.1 × 10^−7^ m^3^ C^−1^ at 2 K.^93^

Resistivity measurements and electron diffraction studies of m-TaS_3_ and o-TaS_3_ showed two transition temperatures (240 K and 160 K), which were interpreted as succeeding Peierls transitions on the diverse chain types of TaS_3_.^94^ For o-TaS_3_, as the temperature dropped below the ambient temperature, the resistance gradually rose up to 230 K and then increased sharply. However, for m-TaS_3_, this happened in a typical manner, *i.e.*, there was a sequential decrease and increase in resistance at 270, 220, and 180 K.^94^ The Peierls transition temperature, the temperature at which the slope of *R vs. T* attains its maximum, was also shown by Roucau *et al.* Peierls transitions in quasi-1D conductors are associated with intrinsic superstructures.^94^ Polarized Raman scattering studies of o-TaS_3_ demonstrated that at the Fermi surface, the formation of a CDW gap was held responsible for the reduction in free carriers.^95^ Thus, the scattering intensity decreased from interband processes along with an increase in the phonon energy. Nanosized TaS_3_ samples showed step-like conductivity as a function of strain, demonstrating the association of the steps with the quantization of the CDW wave vector.^96^ In o-TaS_3_, the asymmetrical conductivity had no dependency below a certain temperature (2 K), which was caused by soliton transport in the CDW system.^97^ Nichols *et al.* studied the frequency and voltage dependencies of voltage-induced torsional strain in o-TaS_3_ and concluded that the strain is allied with a divergence in the CDW instead of the CDW current.^98^ A change in the length, *L*, (depending on the electric field and time) of TaS_3_ samples demonstrated that hysteresis partly corresponds with resistance.^99^ Thermal expansion studies of o-TaS_3_ crystals showed the uncharacteristic behaviour of the hysteresis loop of length *L* below the Peierls transition temperature and at 100 K, the elastic modulus meets the Young's modulus of the CDW wave.^100^ Gorlova and co-workers observed electric-field-induced torsional strain corresponding to colossal shear in TaS_3_ whiskers.^101^ The threshold and hysteresis behaviour of torsion demonstrated its link with CDW deformation. Correspondingly, Nichols *et al.* investigated the effects of hysteretic voltage-induced torsional strain on CDW depinning in o-TaS_3_ using square-wave and triangular-wave voltages of dissimilar frequencies and amplitudes.^102^ Inagaki *et al.* reported the magnetoresistance of a CDW in o-TaS_3_ whiskers under a magnetic field up to 4.2 K. When the field was aligned with the *a*-axis, the maximum amplitude of angle-dependent magnetoresistance was achieved, whereas there was zero value at the *b*-axis.^103^ Electrical transport measurements of single o-TaS_3_ by Farley *et al.* discovered the depression of the Peierls transition temperature to 205 K. Below this temperature, there was depinning of the CDW, which was accredited to broadening of the electric field and surface confinement; low dimensionality and a limited size effect caused a great enhancement in the threshold voltage for the nucleation of CDW dislocations (Fig. 6).^104^ Wu *et al.* studied the pressure-dependent vibrational properties, which imply that the one and only S_‖_ mode (at 54 cm^−1^) was held accountable for the crystalline orientation of TaS_3_ through angle-resolved Raman spectroscopy and high-pressure diamond anvil cell studies.^105^ Frequency-dependent conductivity measurements showed the Drude type behaviour of the inertial feedback of TaS_3_, associated with damping.^106^

**Fig. 7:**
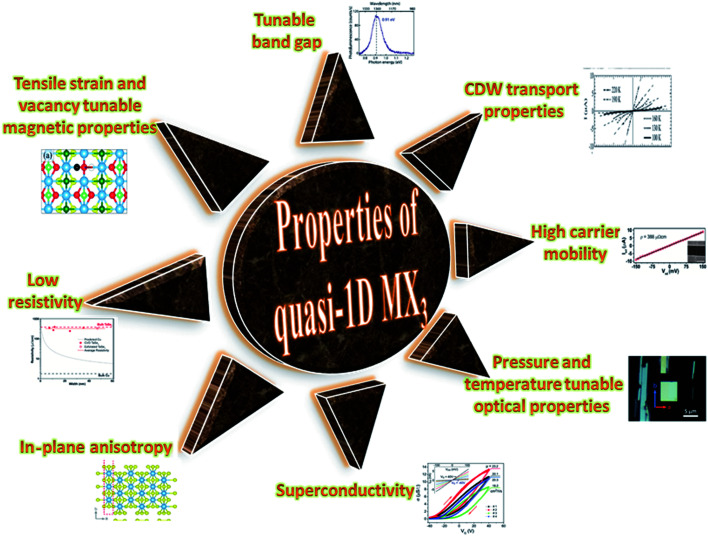
A schematic diagram showing the properties of quasi-1D MX_3_. Resistivity figure reprinted with permission from ref. 78, copyright: 2019, American Chemical Society; in-plane anisotropy figure republished with permission from ref. 9, permission conveyed through Copyright Clearance Center, Inc. (2016); superconductivity figure reproduced with permission from ref. 124, copyright: 2015, Royal Society of Chemistry; optical properties figure reproduced with permission from ref. 6, copyright: 2019, IOP Science; carrier mobility figure reprinted with permission from ref. 78, copyright: 2019, American Chemical Society; CDW properties figure republished with permission from ref. 104, permission conveyed through Copyright Clearance Center, Inc.; band gap figure reproduced with permission from ref. 6, copyright: 2019, IOP Science; magnetic properties figure reproduced with permission from ref. 9, copyright: 2015, American Chemical Society.

TaSe_3_ is reported to be superconducting below 2.1 K with anisotropic properties under the application of a magnetic field^79^ and Kikkawa *et al.* also indicated the superconductivity of TaSe_3_ at *T* = 1.9 K.^65^ Thickness-dependent work function variations of TaSe_3_ flakes confirmed the extended screening length (24 nm) at which the work function suddenly decreased, which specified its hydrophilic nature, and the Raman modes at 128 cm^−1^; 141, 164, 217, and 237 cm^−1^; 177 cm^−1^; and 186 cm^−1^ corresponded to B_g_, B_2_, A_g_, A_1g_ modes, respectively.^107^ TaSe_3_ is reported to have a high current-carrying capacity and high current density, and it has emerged as a possible potential candidate to act as an interconnector in electronic devices due to capping with hexagonal boron nitride (h-BN).^108^ Experimental observations demonstrated that quasi-1D TaSe_3_ nanowires transmit lower levels of normalized noise spectral density, with potential for rationalized local interdependent applications.^109–111^ The main properties of MX_3_ are summed up in Fig. 7.

## 3. Strategies for the growth of MX_3_

Synthesis approaches used for the growth of MX_3_ crystals and nanostructures such as nanowhiskers, nanoribbons, nanosheets, nanowires, *etc.* can be classified as top-down or bottom-up approaches. In this review, we have emphasized different synthesis approaches reported for MX_3_ development, as summarized in Table 3. Useful methods for MX_3_ include direct chemical reactions, chemical vapour transport (CVT), chemical vapour deposition (CVD), high-pressure evolution approaches, intercalation, mechanical and chemical exfoliation, *etc.* Characterization techniques such as X-ray diffraction (XRD), X-ray photoelectron spectroscopy (XPS), field-emission scanning electron microscopy (FESEM), atomic force microscopy (AFM), scanning tunnelling microscopy (STM), transmission electron microscopy (TEM), Raman spectroscopy, UV spectroscopy, and optical microscopy (angle-resolved as well as pressure- and temperature-dependent methods) are the usual procedures used to estimate the properties of MX_3_ materials and further help in exploring their abundant applications.^112–120^

**Table 3:** A list of the various constraints required to grow MX_3_ crystals and nanostructures

Material	Starting precursors	Growth conditions	Heating temperature; substrate	Size	Ref. no.
TiS_3_ crystals	Ti sheets, S powder	Vacuum-sealed (10^−6^ torr) ampoule, 5 days	520 °C; wall of quartz tube	Bulk crystals	24
TiS_3_ thin film	TiCl_4_, *t*-butyl disulfide	Vacuum heating (10^−1^ torr)	260 °C; glass, and Ti and Al foil	Large-area thin film	127
TiS_3_ nanowhiskers	Ti metal foil, S powder	Vacuum-sealed (10^−5^ torr) ampoule, 3 days	500 °C; Ti foil and wall of quartz ampoule	*l* = 100 μm, *w* = few μm, *t* = 0.5 μm	28 and 124
TiS_3_ nanoribbons	Ti disc, S powder	Vacuum-sealed (10^−3^ torr) ampoule, 20 h	520 °C; wall of quartz tube	*l* = 100 μm, *w* = 1–5 μm, *t* = 5–200 nm	144
ZrS_3_ flakes	Zr metal sheets, S pellet	Vacuum-sealed (10^−6^ torr) ampoule, 5 days	650 °C; wall of quartz tube	Bulk needles and mechanically exfoliated flakes	25 and 144
ZrSe_3_ crystals and nanobelts	Zr powder, Se powder	Vacuum-sealed (10^−2^ torr) ampoule, 24 h	650 °C; wall of quartz tube	*l* = tens of μm, *w* = 20–2600 nm	129
ZrTe_3_ crystals	Zr powder, Te powder	Vacuum-sealed ampoule	950 °C (source)/850 °C (sink) and 735 °C (source)/660 °C (sink); wall of quartz tube	*l* = 5 mm, *w* = 0.8 mm, *t* = 0.2 mm	15
HfS_3_ crystals and flakes	Zr metal sheets, S pellet	Vacuum-sealed (10^−6^ torr) ampoule, 5 days	650 °C; wall of quartz tube	Bulk needles and mechanically exfoliated flakes	24
HfSe_3_ crystals and flakes	Hf powder, Se powder	Vacuum-sealed (10^−2^ torr) ampoule, 24 h	650 °C; wall of quartz tube	*l* = tens of μm, *w* = 20–2600 nm	129
HfTe_3_ crystals and flakes	Hf powder, Te powder	Vacuum-sealed, CVT-iodine carrier	500–540 °C; wall of quartz tube	*l* = 0.3 mm, *w* = 0.3 mm, *t* = 0.1 mm	51
NbS_3_ whiskers and flakes	Nb powder, S powder	Vacuum sealed (10^−5^ torr) ampoule, 7 days	550 °C (source)/470 °C (sink); wall of quartz tube, mechanical exfoliation	Whisker-like crystals	84
NbSe_3_ crystals	Nb powder, Se powder	Reaction under vacuum, 15 days	700 °C; wall of quartz tube	*l* = 7.0 mm, *w* = 0.05 mm, *t* = 0.01 mm	87
NbSe_3_ nanoribbons	Nb powder, Se powder	Reaction under vacuum, >15 days	700 °C; wall of quartz tube	*l* = >10 μm to few mm, *w* = *t* = 20–700 mm	72
TaS_3_ crystals and flakes	Ta powder, S powder	Vacuum sealed, CVT-iodine carrier, 48 h	760 °C; wall of quartz tube	*l* = several cm, *w* = 40–900 nm, *t* = 20–50 mm	132
TaSe_3_ crystals and flakes	Ta powder, Se powder	Vacuum sealed, CVT-iodine carrier, 10 days	700–600 °C; wall of quartz tube	*l* = >10 mm, *w* = *t* = ∼200 μm	107 and 108
TaSe_3_ nanowires	TaCl_5_, Se powder	CVD growth under vacuum, Ar + H_2_ gas, 15 min	400 °C; wall of quartz tube and substrate	*l* = >120 nm	78

### 3.1. Bottom-up approaches

#### 3.1.1. IV–X-type MX_3_

**Fig. 8:**
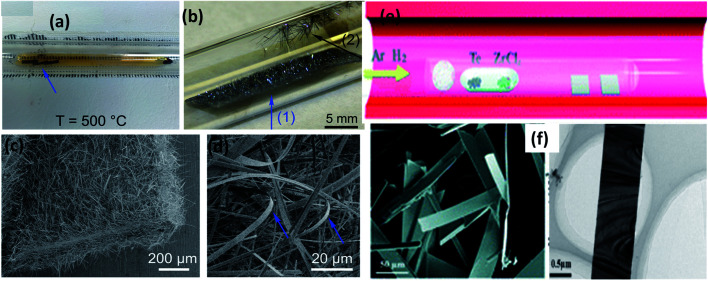
The synthesis of MX_3_. Optical photographs of the ampoule (a) before and (b) after the growth of TiS_3_ whiskers on Ti foil and the surface of quartz. (c and d) SEM images of the grown TiS_3_ whiskers with arrow marks indicating samples collected from the respective positions. Reproduced with permission from ref. 124, copyright: 2015, Royal Society of Chemistry. (e) The synthesis of ZrTe_3_*via* a chemical vapour deposition approach. (f) FESEM and TEM images of ZrTe_3_. Republished with permission from ref. 130, permission conveyed through Copyright Clearance Center, Inc.

Transition-metal trichalcogenides nanostructures, such as nanowhiskers, nanoribbons, and nanosheets, are reported to be grown based on direct reactions involving titanium and sulphur.^2,121–126^ In a typical reaction process for the growth of TiS_3_ crystals and nanostructures, Ti and S are sealed in an evacuated quartz ampoule and heated up to 500–600 °C for several days (3–5 days).^124^ In this approach, the sulfurization temperature and gradient used usually are ∼500 °C and 50–100 °C, respectively. This method is also termed as a chemical vapour transport (CVT) technique, which is an excellent growth mechanism for fabricating low-dimensional materials, where sulphur, TiS_*x*_ species, and sometimes iodine are considered as transport agents.^2^Fig. 8^124^ shows photographs of ampoules with Ti foil and S powder used for the direct reaction-based method to grow bare and doped TiS_3_ nanowhiskers. Fig. 8c and d^124^ show SEM images of nanowhiskers grown on Ti foil and the walls of the quartz tube.^124^ It is reported that the critical temperature for the CVT growth of TiS_3_ should be below a typical temperature, *i.e.*, 632 °C, which is the decomposition temperature of TiS_3_ to TiS_2_ (Table 3). TiS_3_ nanoribbons and nanosheets have been fabricated *via* mixing Ti powder with sulphur gas through a solid–gas reaction. The sulphur powder was heated in a vacuum sealed ampoule at 550 °C (nanoribbons) and 400 °C (nanosheets) for around 15 days.^27^ TiS_3_ thin films are prepared *via* thermal chemical vapour deposition through reacting TiCl_4_ and *t*-butyl disulphide (TBDS) at 260 °C.^127^ The temperature and pressure of the chamber, sulfurizing source, and flow rates of Ar and TiCl_4_ play crucial roles in achieving the formation of pure TiS_3_ films. Similar to the CVT growth approach for TiS_3_, ZrS_3_ and HfS_3_ can also be grown *via* taking Zr and Hf elemental sheets and sulphur powder and heating them in vacuum sealed quartz ampoules at 650 °C for 5 days.^24,128^ ZrS_3_ bulk powder is reported to form upon heating zirconium and sulphur powder in an evacuated quartz ampoule for 150 h, and a stable colloidal dispersion could be formed in different organic media, such as iso-propanol, acetonitrile, ethanol, and dimethyl formamide, *via* a liquid exfoliation process.^35^ ZrSe_3_ and HfSe_3_ nanobelts could be grown *via* a CVT approach, in which Zr or Hf powder was mingled with Se powder at a stoichiometric ratio (1 : 3).^129^ The powder mixture was vacuum sealed (10^−2^ Pa) in a quartz tube and heated at 650 °C for 24 h to achieve the growth of ZrSe_3_ and HfSe_3_ nanobelts. ZrTe_3_ nanoribbons could be grown *via* a CVD approach, in which Te and ZrCl_4_ powder are used as vapour sources (Fig. 8e and f).^130^ Through controlling the carrier gas flow (Ar: 30 sccm and H_2_: 35 sccm), heating temperature (650–750 °C), and growth time (20–80 min), high-quality and single-crystal nanoribbons could be grown on a SiO_2_/Si substrate placed downstream of the quartz tube.^130^

#### 3.1.2. V–X-type MX_3_

**Fig. 9:**
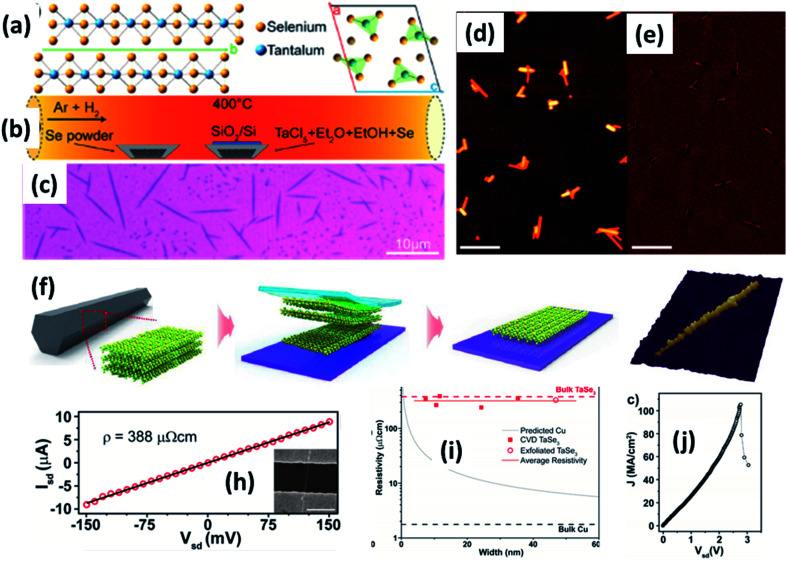
(a) The crystallographic structure of TaSe_3_. (b) A schematic diagram of the CVD set-up for the growth of TaSe_3_ nanowires. (c) An optical image and (d and e) SEM images of a population of TaSe_3_ nanowires. Reprinted with permission from ref. 78, copyright: 2019, American Chemical Society. (f) A schematic illustration of the mechanical exfoliation of TaSe_3_ flakes from bulk TaSe_3_. (g) A 3D representation of a quasi-1D TaSe_3_ nanoribbon monolayer on a SiO_2_/Si substrate. Reproduced with permission from ref. 107, copyright: 2019, Multidisciplinary Digital Publishing Institute. (h) Source-drain current *vs.* voltage for a 11.6 nm TaSe_3_ nanowire in a 2-electrode configuration, with the inset showing an SEM image of the device. (i) Resistivity as a function of bundle width of CVD-grown TaSe_3_ nanowires and a comparison with bulk Cu and exfoliated TaSe_3_. (j) The current density response of a 7 × 7 nm^2^ nanowire as a function of *V*_ds_ with failure at a current density of 108 A cm^−2^. Reprinted with permission from ref. 78, copyright: 2019, American Chemical Society.

The growth of NbX_3_- and TaX_3_-based transition metal trichalcogenides has been reported using similar CVT approaches as followed for the IV–X MX_3_ group, and some of the results are summarized in Table 3. Whisker-like NbSe_3_ nanowires were prepared *via* the direct reaction of a Nb and Se powder mixture placed in a small alumina crucible, sealed under vacuum and heated for 24 h.^89^ Hor *et al.* reported an effective single-step approach for the synthesis of NbSe_3_ nanowires and nanoribbons.^72^ In this approach, stoichiometric quantities of Nb and Se were sealed and heated in a quartz ampoule at 630–700 °C to achieve the growth of pure and crystalline nanoribbons.^89^ Pham *et al.* reported a facile method to prepare few-to-single chain structures of NbSe_3_, encapsulated in BN or CNT sheaths to prevent oxidation.^131^ In this approach, Nb and Se powder was mixed with cap-opened CNTs/BNNTs and vacuum sealed at 10^−1^ torr in a quartz ampoule, followed by heating at 690 °C for 5–9 days. Wu *et al.* reported the preparation of TaS_3_ nanobelts at 760 °C for 48 h in a quartz ampoule *via* a CVT approach, with iodine as the transport agent.^132^ Single crystals of o-TaS_3_ are reported to be formed *via* a CVT approach upon heating Ta sheets and S powder in a sealed quartz tube at 530 °C for a few weeks.^103^ Long nanofibers of o-TaS_3_ can be prepared *via* heating Ta and S precursors in a sealed quartz ampoule at 500 °C for 48 h.^134^ Farley *et al.* prepared o-TaS_3_ nanoribbons *via* heating Ta foil and S powder at 550 °C for 2 h in a vacuum-sealed tube.^104^ The sublimed sulphur travelled across the tube due to a temperature gradient inside the tube and reacted with TaS_3_. o-TaS_3_ whiskers were prepared *via* a CVT approach in a quartz ampoule through maintaining two heating zones at 650 °C and 550 °C in a tube furnace for 7 days.^105^ In a similar approach, Li *et al.* reported the generation of large-scale and self-supported TaS_3_ nanowires *via* the direct reaction of Ta and S powder in a sealed quartz tube at 650 °C for 1 h.^134^ To prepare TaSe_3_ crystals, Ta and Se precursors are heated under vacuum at a pressure of 1 GPa at 500–700 °C for 30–240 min.^135^ Stolyarov *et al.* followed a two-step CVT approach; in the first step, a Ta, Se, and iodine mixture was heated in a quartz ampoule at 900 °C for 12 h, and in the second step, the product was heated in a tube furnace with a temperature gradient of 700–680 °C.^108,110^ In the CVD approach, TaCl_5_ and Se powder samples were used as the reactants and heated at 400 °C for 15 min with Ar and H_2_ as ambient process gases to grow TaSe_3_ nanowires on SiO_2_/Si placed upstream near an alumina crucible filled with TaCl_5_ (Fig. 9a–e).^78^ In another work, Kim *et al.* prepared single-crystal TaSe_3_*via* a CVT approach, mixing stoichiometric amounts of Ta, Se, and iodine powder and heating this at 670 °C for 10 days, maintaining the growth zone at 600 °C, and later mechanically exfoliating it from bulk TaSe_3_ (Fig. 9f–g).^107^ Empante *et al.* reported low resistivity and a high collapse current density of 10^8^ A cm^−2^ for a single nanowire, which featured an electro-migration energy barrier twice that of Cu (Fig. 9h–j).^78^

### 3.2. Top-down approaches

Chemical and mechanical exfoliation methods are widespread synthesis tactics that have gained huge interest in the field of top-down approaches.^137,138^ Mostly, as-prepared bulk crystals are taken as a prime precursor and subsequent chemical intercalation or power-driven force approaches are applied to these. Then, through force or intercalation, the neighbouring layers get detached from adjacent layers. Weak van der Waals forces co-existing between neighbouring layers plays a vibrant role in the materialization of few- to mono-layer crystals of 2D materials upon controlling the relevant parameters. Similarly, the ultrasonication of bulk powder or crystals in solvents is widely used for liquid exfoliation to achieve single- to few-layered nanosheets.

Mechanical exfoliation is a widely used method to isolate single- to few-layered MX_3_ nanosheets from bulk material.^6^ On the other hand, liquid phase exfoliation is an effective method for the large-scale production of MX_3_ nanosheets.^139^ Few-layer TiS_3_ material was prepared by Island *et al.* at a temperature of 400 °C with sheet-like morphology, and this was later mechanically exfoliated to few-layer form.^6^ Xie *et al.* reported the liquid phase exfoliation of bulk ZrS_3_ in *n*-propylamine for 30 min followed by heating at 120 °C for 3 days in a Teflon-lined autoclave.^139^ The obtained product was washed and ultrasonicated in 1-cyclohexyl-2-pyrrolidinone to achieve ZrS_3_ nanosheets. NbS_3_ and NbSe_3_ nanoparticle colloidal solutions were prepared *via* a top-down approach through the ultrasonication of powder in different solutions (DMF, acetone, acetonitrile, ethanol, a water–ethanol mixture, *etc.*) for 3 h. Li-ion intercalation is proposed as a suitable method to prepare MX_3_ nanoribbons, in which three lithium atoms are incorporated into an MX_3_ unit to yield Li_3_MX_3_.^140^ A mechanical exfoliation approach is employed to prepare few-layered flakes from TaSe_3_ crystals (Fig. 9f and g).^107^ A schematic diagram of the different steps used in this process is shown in Fig. 9, in which wafer dicing tape is used and stuck onto the crystal, which is then removed and adhered to the SiO_2_/Si substrate.

## 4. Advanced applications of MX_3_

### 4.1. FETs

**Fig. 10:**
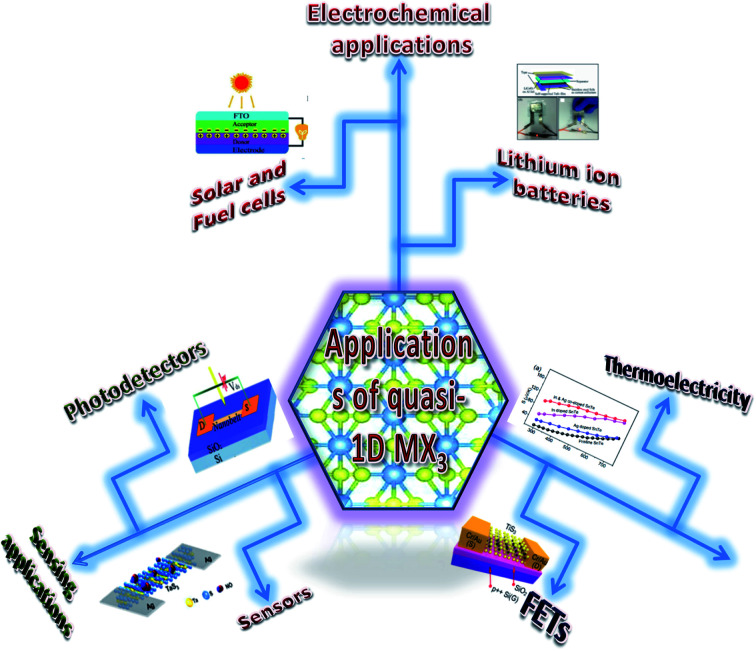
A schematic diagram showing the various applications of MX_3_. Solar and fuel cell figure republished with permission from ref. 33, permission conveyed through Copyright Clearance Center, Inc.; lithium-ion batteries figure reprinted with permission from ref. 134, copyright: 2015, American Chemical Society; sensors figure reprinted with permission from ref. 133, copyright: 2018, American Chemical Society; photodetectors figure republished with permission from ref. 129, permission conveyed through Copyright Clearance Center, Inc.; FETs figure reproduced with permission from ref. 124, copyright: 2015, Royal Society of Chemistry; thermoelectricity figure republished with permission from ref. 157, permission conveyed through Copyright Clearance Center, Inc.; central figure reproduced with permission from ref. 6, copyright: 2015, Springer Nature.

**Fig. 11:**
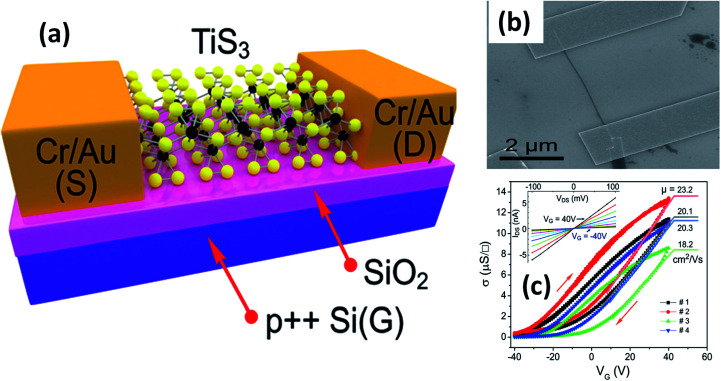
Few-layered TiS_3_ FETs. (a) A schematic diagram and (b) an SEM image of a typical FET. (c) Conductivity *vs.* gate voltage dependencies of four different fabricated FETs, with the inset showing drain source current *vs.* drain source voltage at different gate voltages. Reproduced with permission from ref. 124, copyright: 2015, Royal Society of Chemistry.

The possible applications of TMTCs are shown in Fig. 10. Due to the direct optical band gap (∼1 eV) and ultra-high response of TiS_3_, it has been used as an appropriate material for field-effect transistors with high gain.^125,126^ FETs based on 2D sheets of TiS_3_ whiskers (mechanically exfoliated few-layered samples) have been fabricated on SiO_2_/Si substrates (Fig. 11a and b).^124^ n-Type electronic transport TiS_3_-based FETs exhibited mobilities of 18–24 cm^2^ V^−1^ s^−1^, and this was improved to 43 cm^2^ V^−1^ s^−1^ upon the addition of another substrate, *i.e.*, Al_2_O_3_, through a conventional atomic layer deposition (ALD) procedure. Similarly, the ON/OFF ratio improved to 7000 from 300 upon the use of Al_2_O_3_ as an alternative substrate (Fig. 11c).^124^ The temperature-dependent transfer curves of TiS_3_ nanowire FETs showed a metal–insulator transition, with a cross-over temperature of 220 K and mobility of 20–30 cm^2^ V^−1^ s^−1^.^141^ To demonstrate the anisotropic electrical properties, a TiS_3_ nanosheet FET device was made up, with electrodes at 30° intervals.^126^ The fabricated FET device worked along the *b*-axis of the TiS_3_ nanosheets, which was determined *via* computing the transfer characteristics across the device between two opposite electrodes at variable angles. A polar plot of current *vs.* voltage showed the high mobility of 80 cm^2^ V^−1^ s^−1^ (*b*-axis) and low mobility of 40 cm^2^ V^−1^ s^−1^ (*a*-axis) of the nanosheets. Transfer characteristics curves showed n-type behaviour, with an ON/OFF ratio of five.^126^ The nanoribbons showed lower mobilities with advanced electric field and optical properties, which later can be tuneable. The multifaceted concentration of sulphur vacancies in samples grown at lower temperature played an important role in creating tuneable properties upon an intensification in the n-type dopant.^126^ The interfacial and contact properties of multi-layered TiS_3_ and metals (Au, Ag, Pd, Pt, Ir, and Ni) have been inspected *via* DFT and the outcomes suggested the absence of tunnelling barriers, demonstrating superior carrier injection abilities from the metal to multilayer TiS_3_ in FET and other electrical devices.^142^ The output characteristics of FETs based on HfS_3_ nanobelts confirmed the p-type semiconducting nature.^142^ Therefore, from the above data, it is quite evident that these materials are first and foremost highly suitable materials for nanoelectronics and optoelectronic devices.

### 4.2. Solar and fuel cell devices

**Fig. 12:**
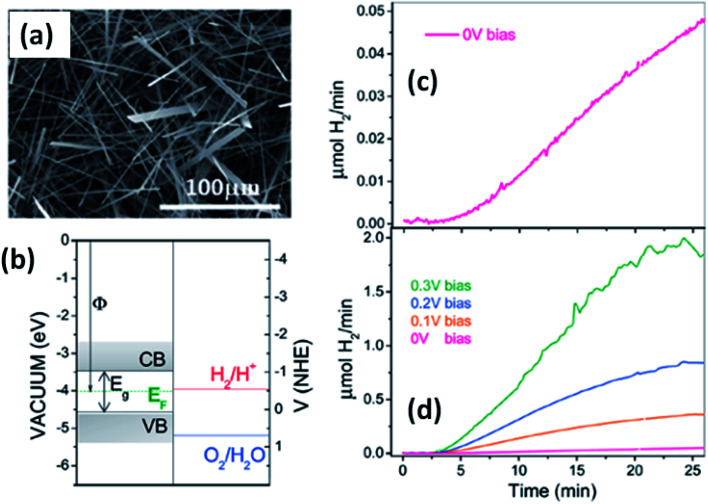
The hydrogen photogeneration properties of MX_3_ nanostructures. (a) An SEM image and (b) the conduction and valence band energy levels on potential (V *vs.* NHE) and energy (eV *vs.* vacuum) scales, with the redox potentials for the water-splitting half reactions at pH = 9.0 *vs.* NHE, of TiS_3_ nanoribbons. The hydrogen evolution flow of TiS_3_ nanoribbons (c) at 0.0 V and (d) at different bias potentials. Republished with permission from ref. 144, permission conveyed through Copyright Clearance Center, Inc.

Solar water splitting using photoelectrochemical cells is an effective and efficient way of storing solar energy in the form of hydrogen, which can be used as a fuel. For hydrogen generation *via* water splitting, the light absorbing material with appropriate energy band positions, *i.e.*, the energy band levels (CB and VB), should be suitable with respect to the water reduction potential.^143,163^ Anisotropic MX_3_ materials possess the advantages of having a low band gap to absorb direct solar energy and being widely available, non-toxic, and suitable for photoelectrochemical water splitting. Fig. 12a^144^ shows an SEM image of TiS_3_ nanoribbons, which have been employed as an active material for electrochemical water splitting.^144^ The systematic energy level band diagram of TiS_3_/electrolyte (Na_2_SO_3_) is shown in Fig. 12b^144^ with a flat band potential (*V*_fb_) at −0.48 *V*_NHE_, and this is used to calculate the semiconductor Fermi level. The conduction and valence band energy levels in terms of potential and energy balance, along with the redox potentials for the water splitting half reactions at pH = 9, are shown in the band diagram. Hydrogen evolution occurs on the TiS_3_ nanoribbons at 0 V and investigations at different bias potentials yielded a photoconversion efficiency of about 7% at a bias potential of 0.3 V (Fig. 12c and d).^144^ In another work, Flores *et al.* demonstrated the energy level schemes for an MX_3_/electrolyte interface, and comparable photogenerated hydrogen fluxes are reported for TiS_3_, ZrS_3_, and HfS_3_.^145^ A TiS_3_ photoanode is reported to generate up to 19 nmol H_2_ per min per cm^2^ at an external bias potential of 0.3 V *vs.* Ag/AgCl. Due to the presence of copious amounts of Se_2_ bonds on the surface of the ZrS_3_ ultrathin nanosheets, enhanced oxygen evolution reaction performance was observed compared to the bulk counterpart.^139^ A low onset overpotential of 244 mV and Tafel slope of 45 mV per decade are achieved using ZrS_3_ nanosheets in strongly alkaline solution (pH = 14), whereas in weakly alkaline solution (pH = 6.9), the onset over-potential and Tafel slope are reported to be lower.^139^

**Fig. 13:**
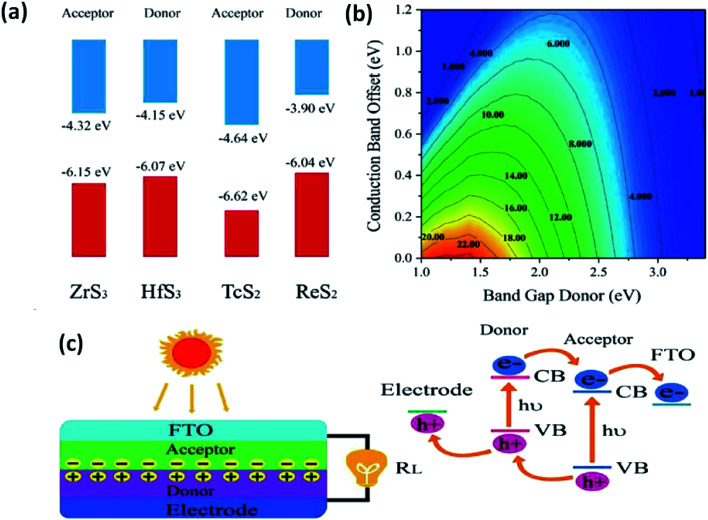
The application of MX_3_ heterostructures to solar cells. (a) Band offsets of ZrS_3_/HfS_3_ and TcS_2_/ReS_2_ with the vacuum level as zero reference. (b) PCE contours obtained as a function of the donor band gap and conduction band offset. (c) A schematic illustration of thin-film solar cells with the associated mechanism. Republished with permission from ref. 33, permission conveyed through Copyright Clearance Center, Inc.

The use of 2D materials and their heterojunctions in optoelectronics and solar-correlated devices is utterly controlled by the superiority of the heterojunction formed and the band alignment to tune carriers at interfaces.^33^ Zhao *et al.* employed DFT to calculate the band superstructures and heterostructures of IV–VIA monolayers of MX_3_ (M = Zr, Hf; X= S, Se) and VIIB–VIA monolayer MX_2_ (M = Tc, Re; X = S, Se). The calculations indicated that for MX_3_, the valence bands are dependent on the p-states of chalcogens, whereas the d-states of the transition metals control the conduction bands. For MX_2_ monolayers, both the valence and conduction bands depend on the d-states of the transition metals. Considering standard water redox potentials (−4.44 eV and −5.67 eV for reduction (H^+^/H_2_) and oxidation (O_2_/H_2_O), respectively), the combination of MX_3_ and MX_2_ monolayers and their band alignment to create efficient heterostructures have been reported.^33^Fig. 13^33^ shows the band-offset components and a contour map of power conversion efficiency (PCE) values. From calculations, it is predicted that a ZrS_3_/HfS_3_ bilayer thin-film device could achieve 16–18% efficiency, which is much higher than other reported 2D heterojunction solar cell devices. Similarly, Ahammed *et al.* reported that the PCEs in ZrS_3_/MoS_2_, ZrS_3_/WS_2_, ZrS_3_/MoSeTe, ZrS_3_/WSTe, and ZrS_3_/WSeTe heterostructured bilayers are as high as ∼12%, 8%, 16%, 14%, and 14%, respectively.^146^ Recently, anisotropic ZrS_3_ has been reported to act as an active material for perovskite light-emitting diodes and P–N junction diodes, and the results are found to be promising, which opens up pathways for further research into the area of MX_3_ heterojunctions for optoelectronics and solar-cell devices.^147,148^

### 4.3. Photodetectors and sensors

**Fig. 14:**
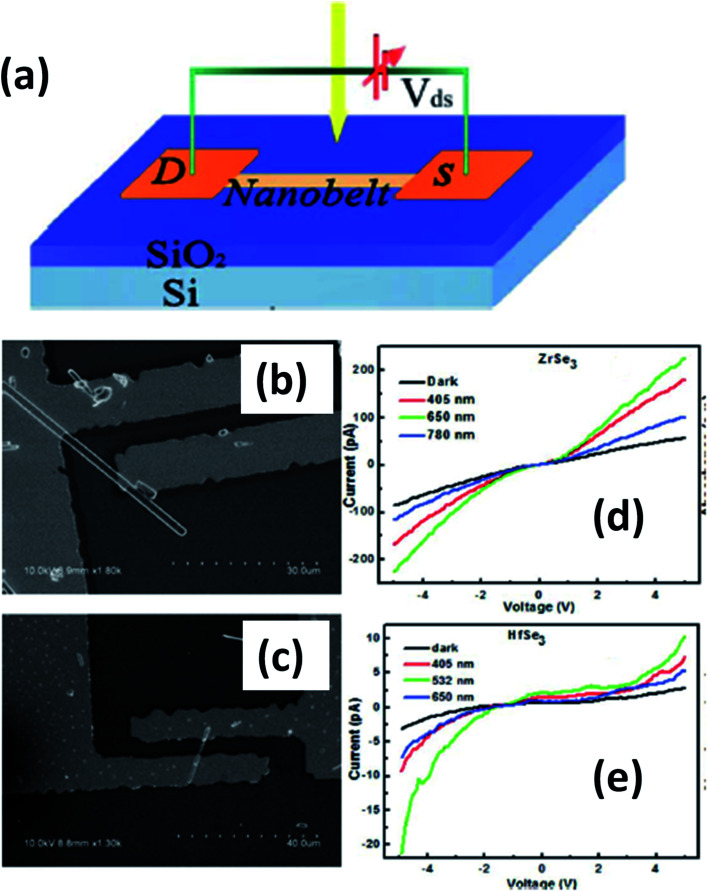
(a) A schematic illustration of a ZrSe_3_ and HfSe_3_ single nanobelt photodetector. SEM images of (b) ZrSe_3_ and (c) the HfSe_3_ photodetector. *I*–*V* characteristics of (d) ZrSe_3_ and (e) the HfSe_3_ nanobelt photodetector. Republished with permission from ref. 129, permission conveyed through Copyright Clearance Center, Inc.

**Fig. 15:**
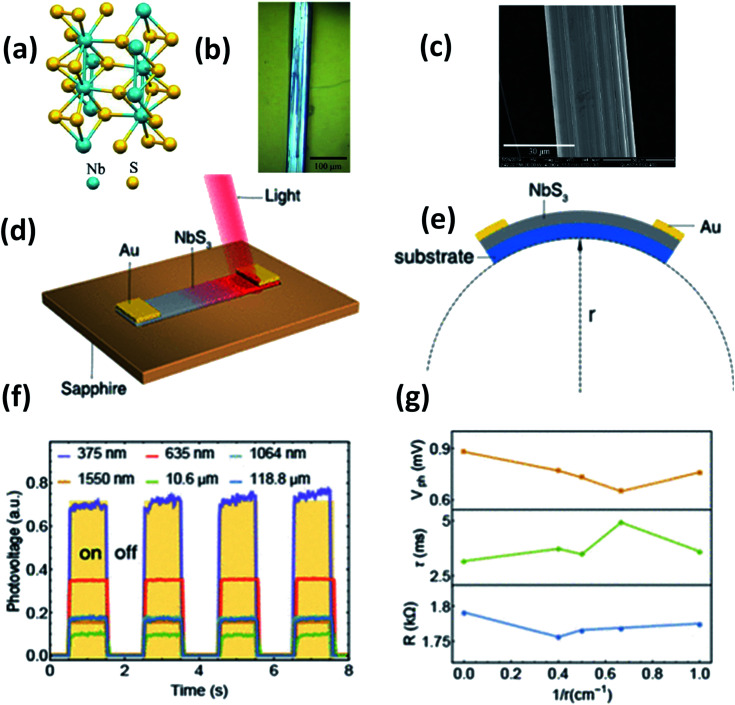
(a) The crystal structure of NbS_3_. (b) Optical microscopy and (c) SEM images of a NbS_3_ crystal. Schematic diagrams of (d) a NbS_3_ photodetector and (e) bending conditions. (f) ON–OFF photovoltage curves at room temperature and (g) resistance, response time, and photovoltage data under different bending conditions. Reprinted with permission from ref. 151, copyright: 2020, American Chemical Society.

**Fig. 16:**
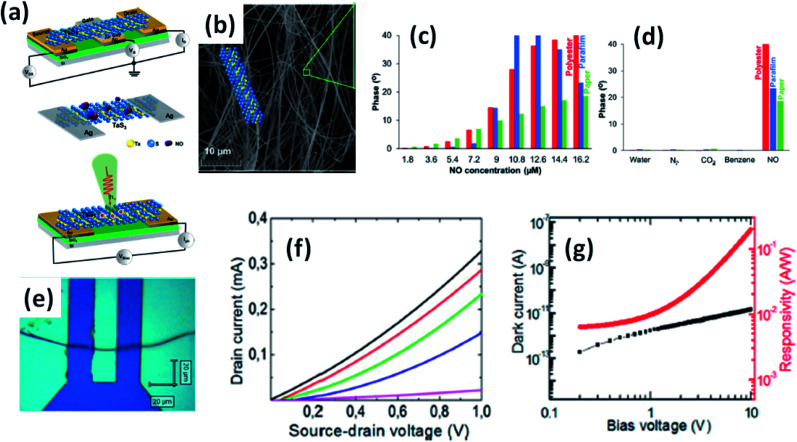
(a) Schematic representations of a FET, NO gas sensor, and photodetector based on TaS_3_ nanofibers. (b) An SEM image of the TaS_3_ nanofibers. (c) The impedance phase responses of gas sensors fabricated on different substrates (polyester, parafilm, paper) as functions of NO concentrations. (d) Selectivity studies of the sensors fabricated on different substrates. (e) An optical image of a FET based on TaS_3_ fibers. (f) FET transistor characteristic curves at different gate voltages (pink: −1 V, blue: 0 V, green: 1 V, red: 2 V, black: 3 V). (g) Dark current (black) and responsivity (red) curves. Reprinted with permission from ref. 133, copyright: 2018, American Chemical Society.

Island *et al.* reported the photoresponse properties of TiS_3_-nanoribbon-based FETs.^125^ TiS_3_ FETs showed a high photoresponse of up to 2910 A W^−1^, and fast switching times of ∼4 ms, with a cut-off frequency of 100 Hz, showing promise for photodetection and photovoltaic applications. Tao *et al.* reported flexible a visible light photodetector formed on ZrS_3_ nanobelt film, which showed high spectral selectivity, a wide range, and a rapid photoresponse in the visible light to near-infrared region.^149^ Photodetectors based on HfS_3_ FETs showed a huge ON/OFF ratio of 337.5, along with an ultralow dark current of 0.04 pA at 405 nm under 1.2 mW cm^−2^ light excitation.^32,142^ CVT-deposited ZrSe_3_ and HfSe_3_ were investigated for photodetection applications.^129^ Under excitation by 650 nm wavelength light, a ZrSe_3_ photodetector showed a light ON/OFF ratio of 1.92 at 50 s with a bias voltage of 5 V (Fig. 14).^129^ Similarly, a HfSe_3_ nanobelt photodetector showed an ON/OFF ratio of 2.2 with an average time of 50 s, and the photoresponse time was 0.4 s. Although HfTe_3_-based materials are reported to show interesting properties, including CDWs, superconductivity, and the ability to be used in quantum Hall-effect-related devices, they have been less studied to date.^51,150^ Wu *et al.* reported a photothermoelectric (PTE) detector based on NbS_3_ with considerable performance in the UV to terahertz range.^150^ Considering its immense surface-to-volume ratio and reduced magnitude, mechanically exfoliated quasi-1D NbS_3_ crystals prepared *via* a CVT approach were studied for photodetector applications (Fig. 15a–d).^151^ Various types of continuous-wave lasers were used to test the photoresponse performance, including UV (375 nm), near-infrared (NIR, 1064 and 1550 nm), visible (635 nm), and semiconductor lasers, a mid-infrared (MIR, 10.6 μm) CO_2_ laser, and a terahertz-wave-generating far-infrared gas laser (118.8 μm) (Fig. 15e).^151^ The photodetector based on NbS_3_ flakes showed good performance, with responsivities higher than 1 V W^−1^, a response time of ∼7 ms, and robust flexibility and stability (Fig. 15e–g).^151^*Via* employing the interesting structural and attractive electronic properties of layered TaS_3_ prepared in the form of nanofibers, Pumera and co-workers reported a highly selective impedimetric NO gas sensor, FET and photodetector (Fig. 16).^133^ The nanofibers showed metallic character, with a metal-semiconducting transition below 210 K, prompted by CDW formation. The impedimetric gas sensor showed an excellent response towards NO when fabricated on different substrates (Fig. 16).^133^ A TaS_3_-based sensor fabricated on a polyester substrate showed the best sensing performance, with a limit of detection (LOD) of 0.48 ppb.

### 4.4. Lithium ion batteries

**Fig. 17:**
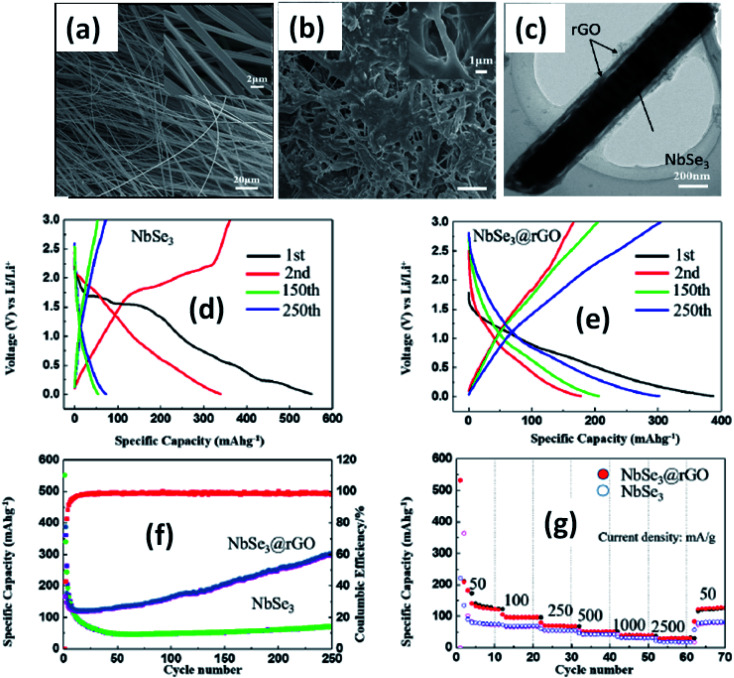
SEM images of (a) NbSe_3_ and (b) NbSe_3_@rGO. (c) A TEM image of NbSe_3_@rGO. The electrochemical performance of NbSe_3_ and NbSe_3_@rGO in the voltage range of 0.005–3 V *vs.* Li/Li^+^. Discharge and charge curves of (d) NbSe_3_ and (e) nanobelts. (f) The cycling performance of NbSe_3_@rGO and NbSe_3_ nanobelts at a current density of 100 mA g^−1^. (g) The rate performances of NbSe_3_@rGO and NbSe_3_ nanobelts. Reprinted from ref. 155, copyright: 2017, with permission from Elsevier.

**Fig. 18:**
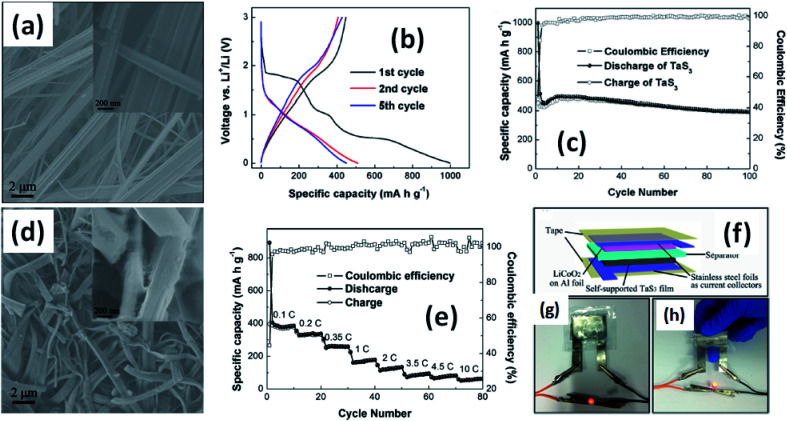
(a) An SEM image of TaS_3_ nanowires. (b) Voltage profiles of a lithium-ion battery based on TaS_3_ nanowires cycled between 0.001 and 3 V *vs.* Li^+^/Li at a cycling rate of 0.1C. (c) Capacity and coulombic efficiency as a function of cycle number for a TaS_3_ nanowire electrode at a cycling rate of 0.1C. (d) An SEM image of nanowires after long-term cycling tests. (e) Rate performance and coulombic efficiency as a function of cycle number for TaS_3_ electrodes and as a function of discharge rate (0.1–10C). (f–h) A schematic diagram of a fabricated flexible lithium-ion battery with photographs showing a glowing LED powered by flat and bent states. Reprinted with permission from ref. 134, copyright: 2015, American Chemical Society.

Due to their multi-electron processes with high theoretical capacity, MX_3_-based materials have emerged as potential active materials for lithium- and sodium-ion battery applications.^152–155^ Wu *et al.* investigated Li/Na adsorption and diffusion in bulk, few-layer, and monolayer TiS_3_*via* studying the phase stability, electron properties, adsorption and dispersion properties, capacity, and plateaus. Charge density and Bader charge analysis studies provided information about the interactions of Li/Na with nearby atoms.^152^ The above-mentioned analysis methods also confirmed significant charge transfer from Li or Na atoms to surrounding sulphur atoms, and this effect was prominent in monolayer TiS_3_. Amorphous TiS_3_, prepared *via* the ball milling of TiS_2_ and S, is testified to show a capacity of 400 mA h g^−1^ when used as a Li-ion battery electrode material.^153^ Tanibata *et al.* reported an all-solid-state sodium cell using TiS_3_ as the active material, which showed a capacity of over 300 mA h g^−1^ during the first charge–discharge process.^154^ The capacity of a cell with a TiS_3_ electrode is reported to be three times higher than that of a cell with TiS_2_ crystals. *Ex situ* characterization (XRD and Raman spectroscopy) studies of the electrode materials after long-term cycling tests confirmed that TiS_3_ maintained its amorphous nature and local structure. Considering the advantages of its metallic nature and the high surface area of NbSe_3_, Li *et al.* demonstrated its application as an anode material in lithium-ion batteries.^155^ Pure and NbSe_3_ nanobelts wrapped with reduced graphene oxide (rGO) were prepared *via* a chemical approach (Fig. 17a–c).^155^ The wrapped rGO utilized strain from NbSe_3_ structural distortion to suppress impairment induced by volume alterations in the structure of NbSe_3_ nanobelts during charge–discharge cycles (Fig. 17g).^155^ NbSe_3_@rGO showed a discharge capacity of 300 mA h g^−1^ after 250 cycles at a current density of 100 mA g^−1^, which was four times greater than that of untainted NbSe_3_ nanobelts. Li *et al.* demonstrated self-supported and flexible lithium ion battery anode electrodes based on TaS_3_ nanowires with a good reversible capacity of ∼400 mA h g^−1^ after 100 cycles at 0.1C with 0.1% decay (Fig. 18).^134^ Due to the continuous and interconnected nature of the TaS_3_ nanowires, binder-free and self-supported electrodes could be fabricated, which not only enabled fast electron and ion access but also provided high mechanical flexibility in the fabricated cells.

### 4.5. Thermoelectricity

The thermoelectric effect is mainly a translation between temperature gradients in a material and electronic voltage, and it also works contrariwise. Thermoelectric constituents convert heat into electric power and they have been used to design eco-friendly and sustainable energy sources.^156,157^ The efficiency of thermoelectric materials is estimated based on the figure of merit parameter, *ZT* = *S*^2^*σT*/*k*, where *S*, *σ*, *T*, and *k* are the Seebeck co-efficient, electrical conductivity, absolute temperature, and thermal conductivity, respectively. The thermal conductivity component includes both electronic (*k*_e_) and phonon (lattice, *k*_l_) parts. Hence, a proper combination of *S*, *σ*, and *k* is expected to lead to high-performance thermoelectric materials. Some strategies, such as using low-dimensional structures, alloy defects, band engineering, *etc.*, have been employed for this purpose.^158–161^ The thermoelectric properties of MX_3_ materials are reported to show robust anisotropic behaviour in bulk, few-layer, and monolayer form.^162^ Wang *et al.* reported that the power factor (*S*^2^*σ*) along the *Y*-direction is increased compared to that along the *X*-direction for ZrS_3_.^162^ Phonon transport findings also demonstrate large anisotropy characteristics induced by unlike scattering from layered ZrS_3_ along the *X* and *Z* directions and different group velocities along the *X* and *Y* directions. DFT studies of ZrSe_3_ monolayers reveal that corrugated conduction levels can result in solid anisotropy of the electric transport characteristics.^30^ It has also been reported that heat transference in ZrSe_3_ monolayers is completely under-controlled by superficial Se atoms. The large bond lengths of the Zr–Se_1_ and Zr–Se_2_ chains confine their influence on heat transference and unswervingly lead to low levels of lattice thermal conductivity.

## 5. Summary and future directions

Thorough experimental and theoretical studies of quasi-1D MX_3_ have shown their wide range of applications in the fields of condensed matter physics and nanotechnology due to their various outstanding electrical, optical, magnetic, and CDW properties, and strong in-plane anisotropy with a quasi-1D nature. The dynamic crystal structures and new fabrication techniques of these materials are well depicted in this work. Also, novel applications based on these materials can be expanded to countless conceivable concepts, which can be viewed perfectly based on the above details.

Weak van der Waals forces of attraction between layers and anisotropy along the chain axis (*b*-axis) provide MX_3_ materials with the advantages of both structural (2D layered material and quasi-1D) and electrical (CDW phenomena) properties. In MX_3_, the chalcogen atoms (S, Se, Te) are considered to be electron reservoirs, thus providing exceptional electronic properties, arising from bulk and nanostructured materials. The M atom presents at the centres of all prismatic chains of MX_6_, which are linked to each other in an infinite chain manner and run parallel to the *b*-axis. At the base of the prism of MX_3_, one bond is shorter compared to the others, so more research needs to be carried out in this direction utilizing the advantages of this property, so that new applications of these materials can be explored. Even TiS_3_ shows strong dichroism in agreement with systematic anisotropy. Similarly, the creation of different types of vacancies in the TiS_3_ system leads to the induction of a definite magnetic moment. Spin–orbit interactions in the case of ZrX_3_ materials result in an increase in the energy split from S to Se atoms. Certain phonon modes undergo a dramatic linewidth reduction near *T*_CDW_, representing the strong coupling of phonons with electronic degrees of freedom associated with the CDW for ZrTe_3_. However, huge attention and focus should be given to the interesting property of MX_3_, *i.e.*, the CDW, so that the shift in the CDW transition can be well-understood and numerous unexplored applications can be instigated in this direction. High-end techniques and better qualitative practical methods, like solid-state NMR, photon correlation spectroscopy, X-ray diffraction topography (XRT), *in situ* and *ex situ* characterization (XRD, SEM, and TEM), muon-spectroscopy, terahertz spectroscopy, *etc.*, can be engaged to study and visualize the LRO pyramid in few-layer or monolayer MX_3_. The properties are quite poor along the *a*-axis, and they can be tuned *via* the application of external pressure, strain, *etc.* More and more focus should be given to studying the anisotropy in the *a*-axis direction, which is somewhat present in some metal tri-tellurides and will be helpful for outstanding applications in the field of nanotechnology. Therefore, these properties open up the endless possibility for spectacular applications. In contrast, 2D layered material characteristics are somehow not properly shown by the materials due to the quasi-1D nature.

Synthesis approaches for MX_3_ materials are quite cost-effective and environmentally friendly and less time-consuming in comparison to other 2D material families, like MXenes. These methods include direct chemical reactions, chemical vapour transport (CVT), chemical vapour deposition (CVD), mechanical and chemical exfoliation, *etc.* Subsequently, other atoms can be intercalated into the layers of MX_3_ in line with the feeble van der Waals forces. Characterization techniques such as X-ray diffraction (XRD), X-ray photoelectron spectroscopy (XPS), field-emission scanning electron microscopy (FESEM), electron-dispersive X-ray analysis (EDAX), atomic force microscopy (AFM), scanning tunnelling microscopy (STM), transmission electron microscopy (TEM), Raman spectroscopy, UV-visible spectroscopy, and optical microscopy are typical methods used to appraise the properties of MX_3_ materials. Mostly, CVD and CVT methods are used for producing bulk as well as nanostructured materials with layers of few mm to nm thickness. As monolayer materials show interesting and research-driven properties, innovative and scalable exfoliation techniques can be used to form thin monolayers.

Most materials are fabricated *via* taking both precursors at a particular stoichiometry, placing them into a vacuum-sealed ampule, and heating them at a certain temperature for a precise amount of time. Later, after the system cools down, the prepared samples are removed. *Via* these methods, nanowhiskers, nanoribbons, nanosheets, *etc.* of MX_3_ (TiS_3_, ZrX_3_, HfX_3_, NbX_3_) are prepared, and their characterization is carried out *via* the above-stated techniques. However, these methods are exceedingly time consuming, and some experiments take 15 days. Therefore, more effective strategies should be implemented for the growth of bulk as well as monolayered MX_3_. Upgraded CVD and CVT systems with high-end programming for the skillful flow of inert gases (Ar, H_2_, N_2_, or a mixture of any two) and proper temperature maintaining tools should be prearranged to control the number of layers and their thickness.

MX_3_ materials show a wide range of applications, *e.g.*, FETs, solar and fuel cells, lithium-ion batteries, photodetectors, photosensors, and thermoelectrics. The higher mobilities and greater ON/OFF ratios of MX_3_-based FETs are worth noting in the present day. MX_3_ materials possess the advantages of having a low band gap to absorb direct solar energy, and being low-cost, plentiful, and appropriate for photoelectrochemical water splitting, making them suitable candidates for use in solar and fuel cells. The maximum efficiency reached is about 16–20%. The high photoresponse, fast switching time, and high cut-off frequency make MX_3_ the best material for use in sensors and photodetectors. MX_3_ can also be used in converting heat into electrical power due to its transport properties. Despite all the merits possessed by MX_3_, these materials are well behind in terms of real-world applications, like in supercapacitors, sodium-ion batteries, *etc.* These materials can be fused with carbon-based materials (single-walled and multiwalled carbon nanotubes and rGO) for supercapacitor applications due to their high surface areas. The efficiencies of solar cells can be improved *via* the incorporation and doping of active elements, which can allow more focus on the real-world applications of MX_3_. Work should be done to increase the capacities of lithium-ion batteries based on MX_3_*via* the strong intercalation of other atoms into the layers of MX_3_. The photoresponse can be enhanced along with the switching time in photodetectors. MX_3_ materials can be used for building biosensors, as they are non-toxic and eco-friendly in nature.

This review article highlights the significance of the in-plane anisotropy and quasi-1D nature of MX_3_ materials, with a complete study of the atomic structures, physical and chemical properties, intrinsic modulations in CDW states, cost- and time-effective synthesis methodologies, and related plausible applications. We contemplate optimistically that this fragment of work on quasi-1D MX_3_ materials, the evolution of their primary properties, and their related applications will have contemporary significance, leading to novel and fresh research.

## Conflicts of interest

There are no conflicts to declare.

## Supplementary Material

RA-010-D0RA07160A-s001
