# Supplementary material for: Anisotropic quasi-one-dimensional layered transition-metal trichalcogenides: synthesis, properties and applications
Source: RSC Adv. 2020 Oct 2;10(60):36413–38. doi: 10.1039/d0ra07160a (PMC9057157; doi:10.1039/d0ra07160a)
Supplement: RA-010-D0RA07160A-s001 [file RA-010-D0RA07160A-s001.pdf]

Electronic Supplementary Material (ESI) for RSC Advances.
This journal is © The Royal Society of Chemistry 2020

Figures

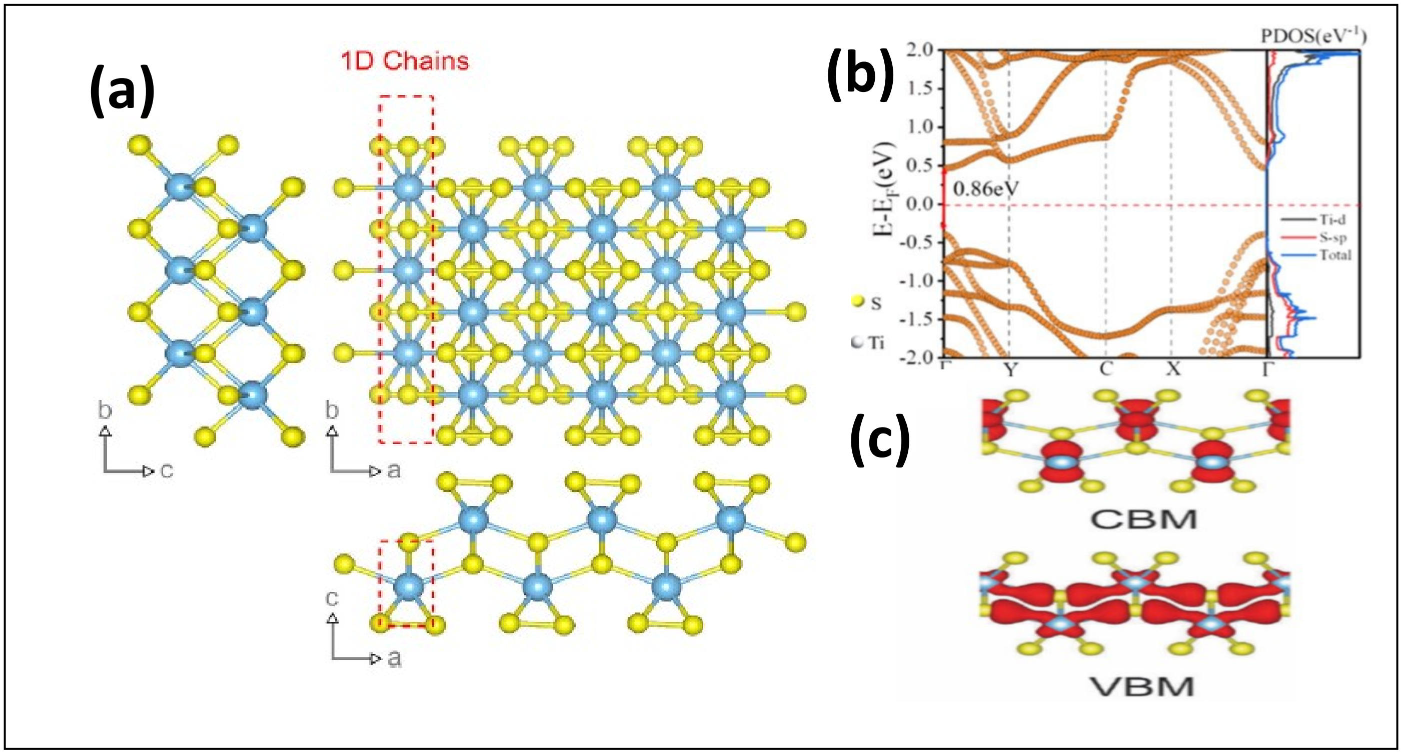

Figure 1: (a) Crystal structure of anisotropic TiS_3 indicating the bond lengths between titanium and sulfur along the b-axis and a-axis with shorter along the b-axis, reproduced with permission from Ref- (6), copyright (2015) Springer Nature (b) Calculated electronic energy band structure along symmetry directions of the Brillouin zone (Γ-Y-C-X-Γ) and the projected density of states (PDOS) of the optimized structure of ML TiS_3. The direct band gap Eg of ML TiS_3 is indicated by red arrow. The Fermi level is set as zero energy (the dashed lines), Reprinted with permission from ref no-8. Copyright (2019) from American Chemical Society (c) The charge distribution of the VBM and CBM states of monolayer TiS_3, republished with permission from ref. no.-9; permission conveyed through Copyright Clearance Center, Inc.

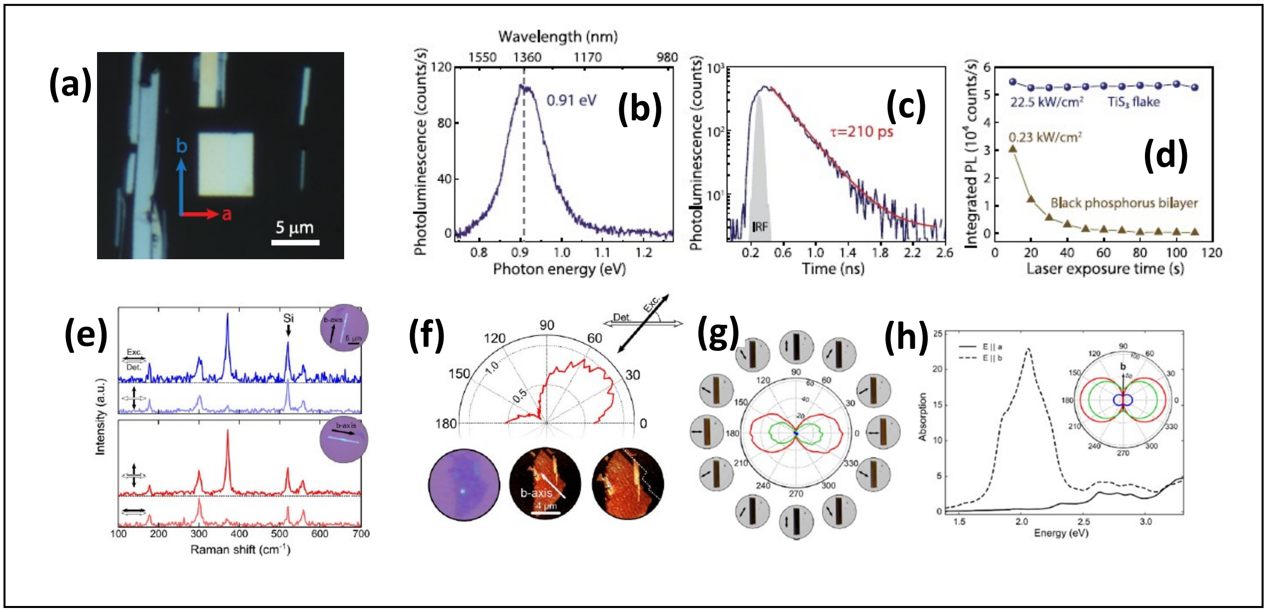

Figure 2: Optical properties of TiS_3 flakes: (a) optical microscope image with a and b axis directions, Infrared light emission from TiS_3 by (b) photoluminescence and (c) time-resolved photoluminescence, (d) phtostability of a TiS_3 flake compared to a black phosphorous, reproduced with permission from Ref- (6), copyright (2019) Iopscience, (e) Raman spectra of a TiS_3 ribbon with horizontal excitation and detection polarization, (f) intensity of the 370 cm^{-1} Raman peak of a 3 nm thick flake (3-4 layers) as a function of the excitation polarization angle, Ref no-18, (g) transmittance of the red, green and blue channels as a function of the excitation polarization angle, (h) calculated absorption spectra when the field is aligned parallel to the b-axis (dashed line) and a-axis (solid line) with the inset showing transmittance in the a-b plane for energies red (1.9 eV), green (2.4 eV), and blue (2.72 eV) excitations, reproduced with permission from Ref- (6), copyright (2015) Springer Nature.

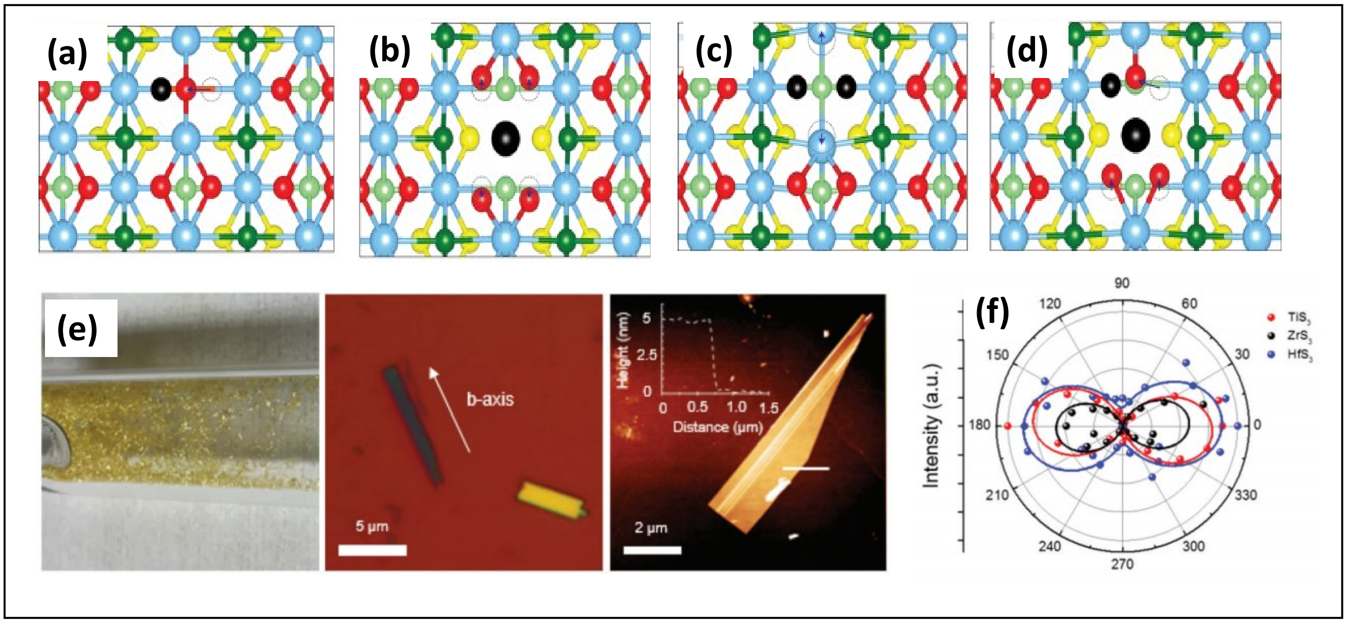

Figure 3: Top view of relaxed monolayer TiS_3 with (a) S vacancy, (b)Ti vacancy, (c) double S vacancy, and (d) TiS vacancy. Black atoms illustrate removed atoms, and dashed circles show initial position of the displaced atom, Reproduced with permission from ref. no-19, copyright (2015) American Chemical Society, (e) Optical photograph of HfS3 needles grown on the inner walls of a quartz ampoule, optical image of ZrS_3 flakes exfoliated onto SiO_2 substrates with the b-axis direction shown, and an AFM image of an exfoliated TiS_3 flake with thickness of ~5 nm. (f) Polar plot of mode III at 372 (red), 320 (black), and 321 cm−1 (blue) that correspond to thin TiS_3, ZrS_3, and HfS_3 flakes respectively, republished with permission from ref. no.-24; permission conveyed through Copyright Clearance Center, Inc.

.

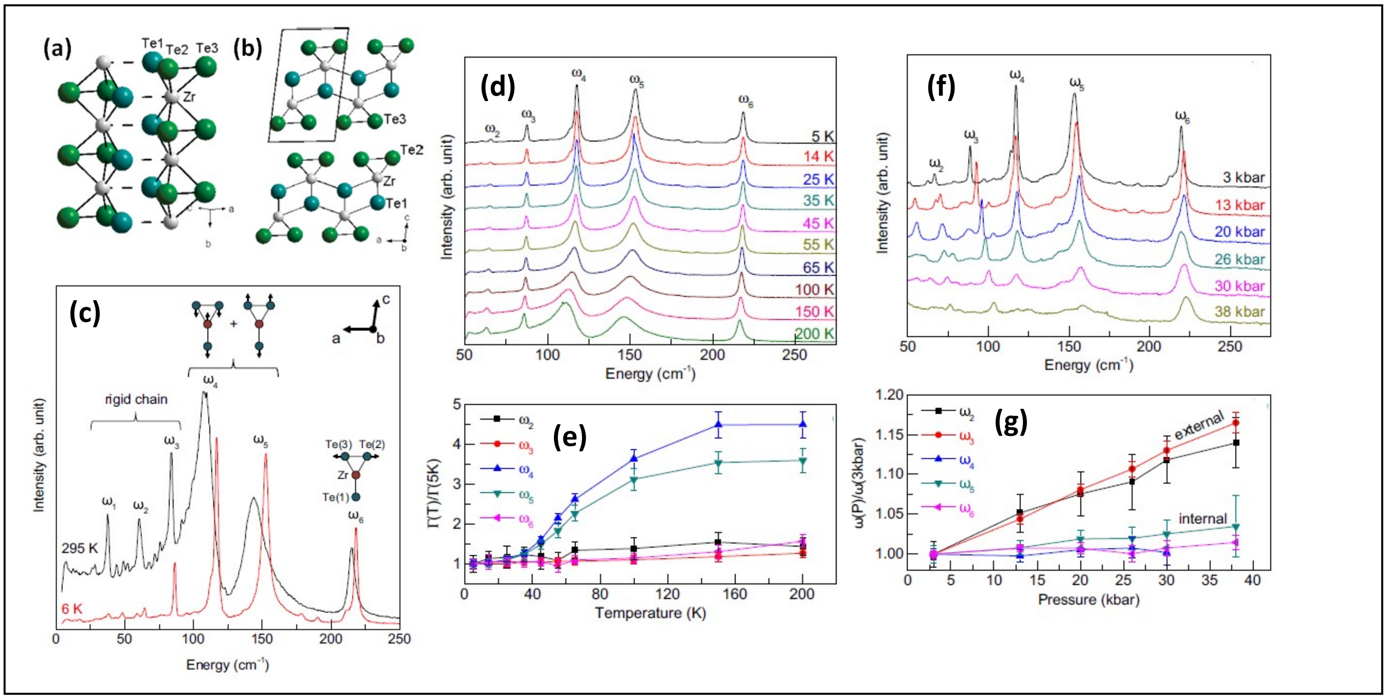

Figure 4: Crystal structure of $ZrTe_3$: (a) The quasi one-dimensional trigonal prism packing along the b axis, (b) quasi two-dimensional $ZrTe_3$ layer along the a-c plane, Reprinted with permission from Ref. no-15, copyright (2013) American Physical Society (c) Raman spectra of $ZrTe_3$ at 6 K and 295 K. (d, e) Temperature dependent Raman spectra at ambient pressure and (f, g) pressure dependent Raman spectra at T=3 K, Reprinted with permission from Ref. no-36, copyright (2013) American Physical Society

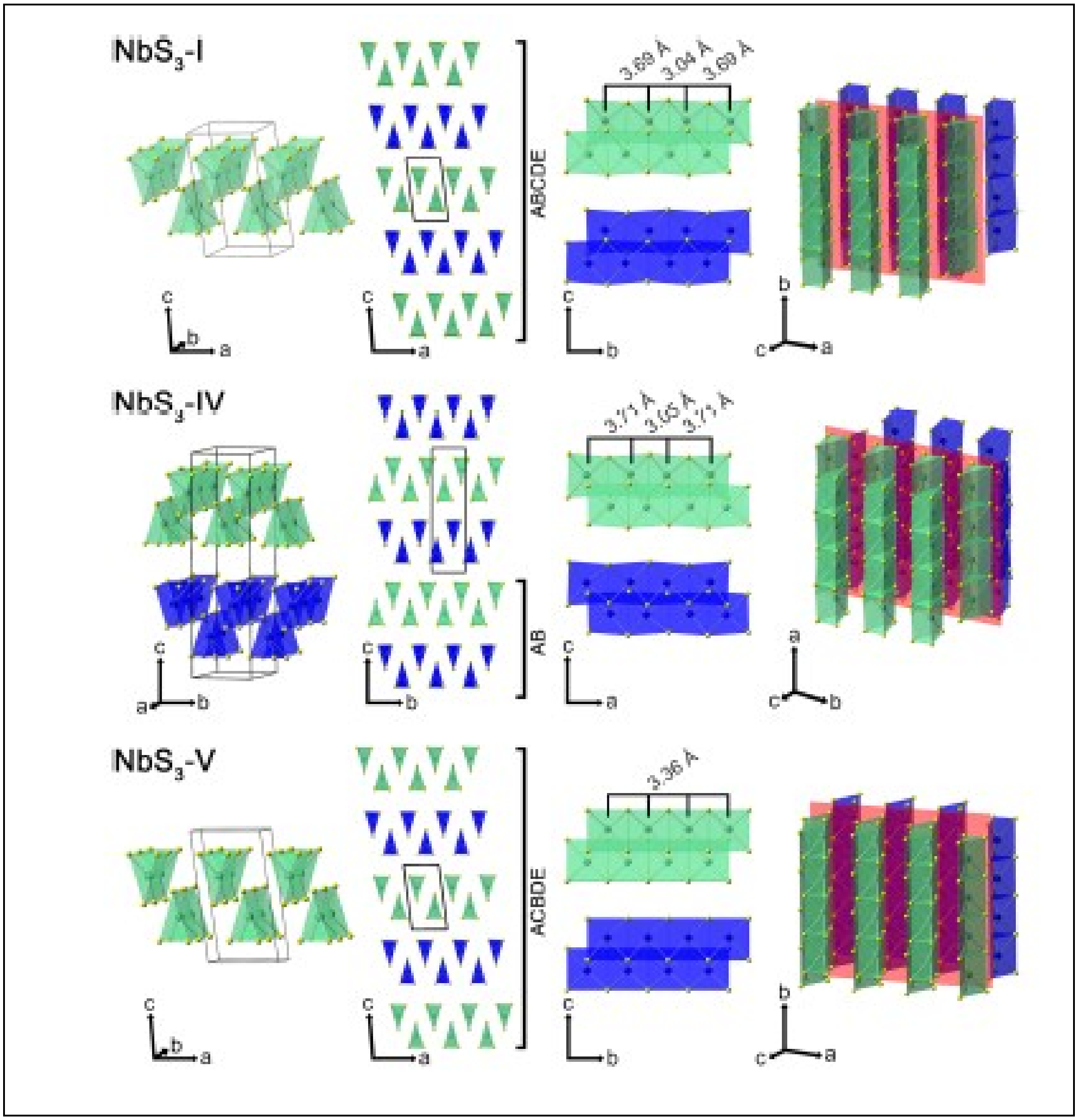

Figure 5: Crystal structure of $NbS_{3\text{-I, IV, V}}$: Unit cell, chain cross section, layer and perspective views, Reproduced with permission from ref. no.-55, copyright (2017) Application Infrastructure Provider**.**

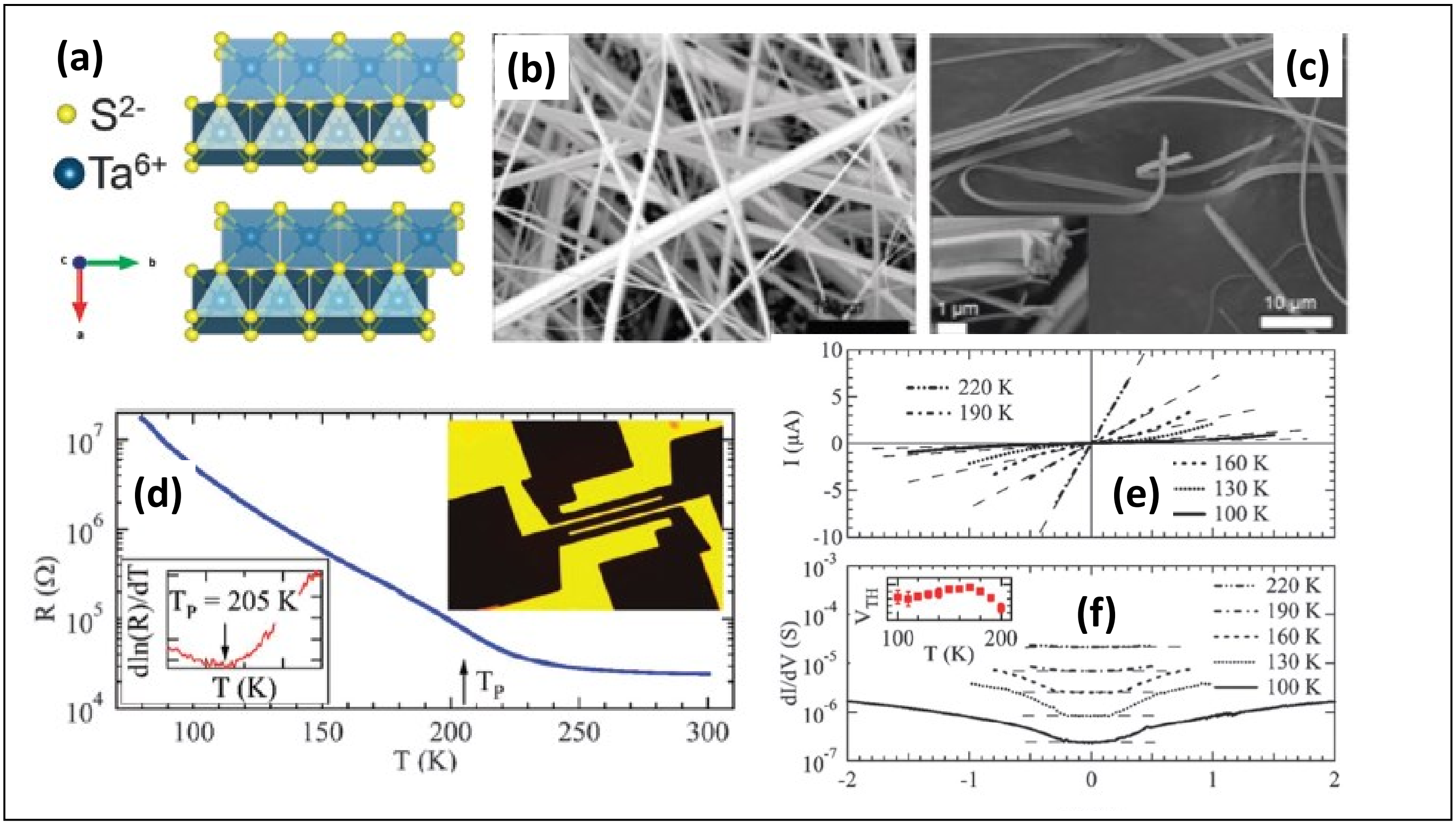

Figure 6: (a) Orthorhombic structure of TaS_3, (b, c) FESEM images of the TaS_3 nanobelts, (d) temperature dependence resistance change of a single nanoribbon with the inset showing the fabricated device, (e) current-voltage curves and (f) differential conductance as a function of voltage measured at specific temperatures with the inset showing temperature dependence threshold voltage, Republished with permission from ref. no.-104; permission conveyed through Copyright Clearance Center, Inc.

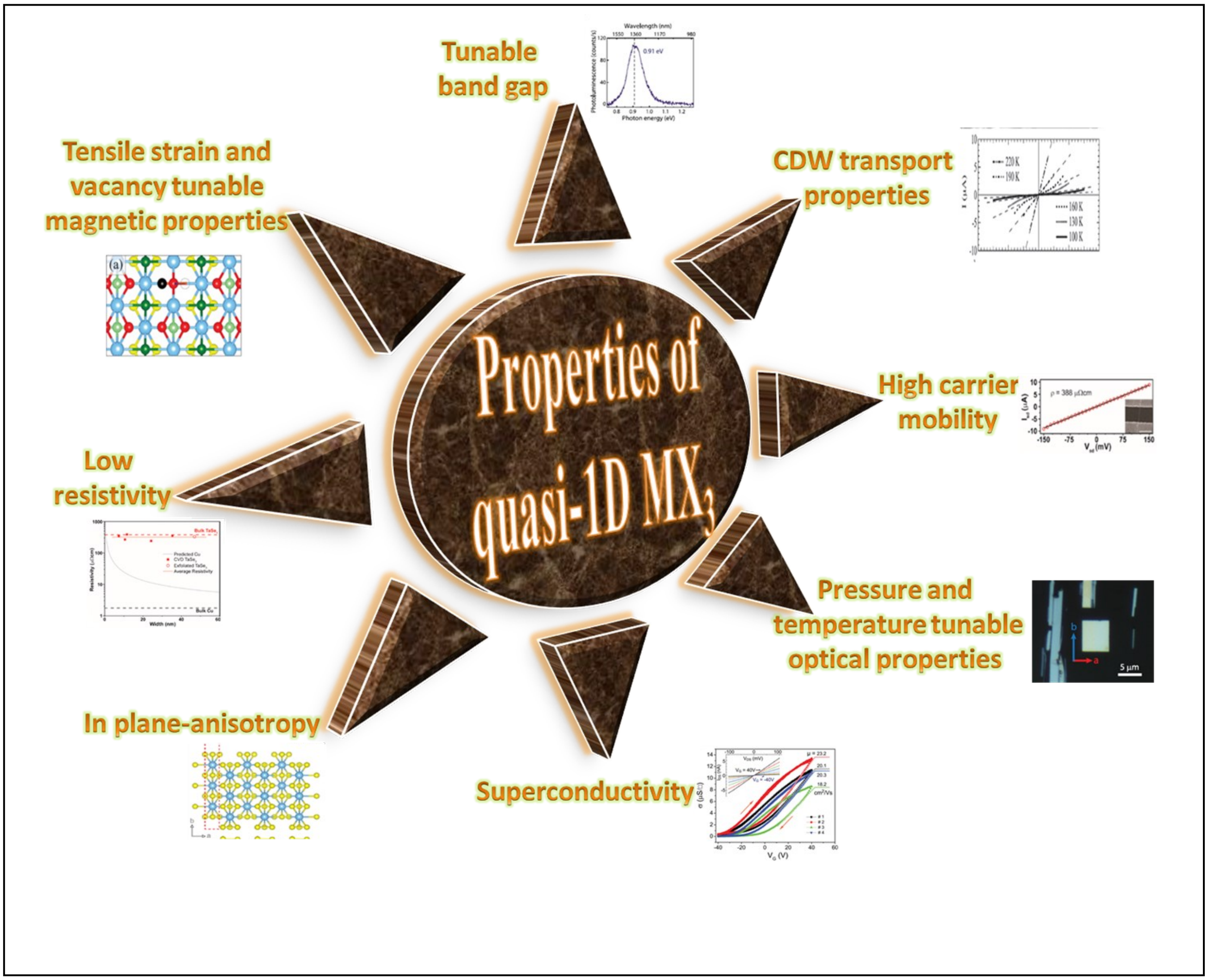

Figure 7: Schematic diagram showing properties of quasi-1D MX_3, resistivity fig.-Reprinted with permission from Ref. no-78, copyright (2019) American chemical Society, in plane anisotropy fig.-Republished with permission of ref. no.-9 permission conveyed through Copyright (2016) Clearance Center, Inc, superconductivity fig.- Reproduced with permission from ref. no.-124, copyright (2015) Royal Society of Chemistry, optical properties- reproduced with permission from Ref- (6), copyright (2019) Iopscience, carrier mobility fig.-Reprinted with permission from Ref. no-78, copyright (2019) American chemical Society, CDW properties-Republished with permission from ref. no.-104; permission conveyed through Copyright Clearance Center, Inc, band gap fig.- reproduced with permission from Ref- (6), copyright (2019) Iopscience, magnetic properties fig.- Reproduced with permission from ref. no-9, copyright (2015) American Chemical Society

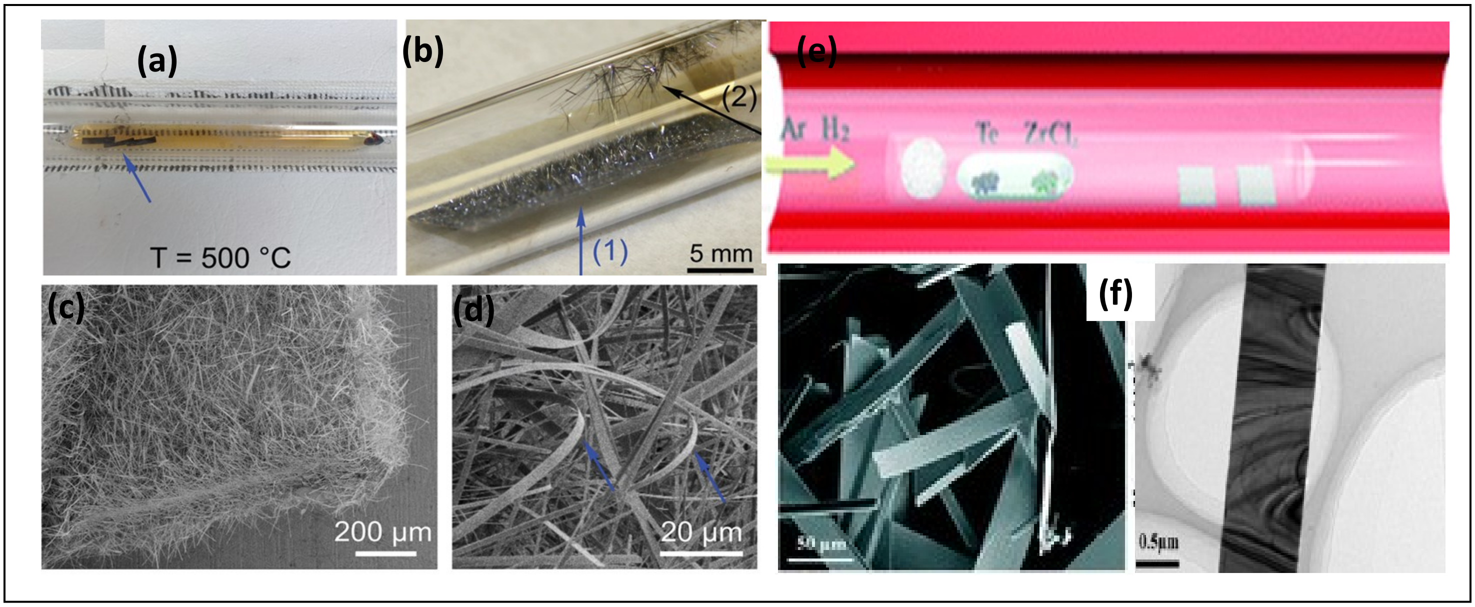

Figure 8: Synthesis of MX_3: Optical photograph of the ampoule (a) before and (b) after the growth of TiS_3 whiskers on Ti foil and surface of quartz. (c, d) SEM images of grown TiS_3 whiskers with arrow marks indicating the samples collected from the respective positions, Reproduced with permission from ref. no.-124, copyright (2015) Royal Society of Chemistry(e) Synthesis of $ZrTe_3$ by chemical vapour deposition approach, (f) FESEM and TEM image of $ZrTe_3$, Republished with permission from ref. no.-130; permission conveyed through Copyright Clearance Center, Inc.

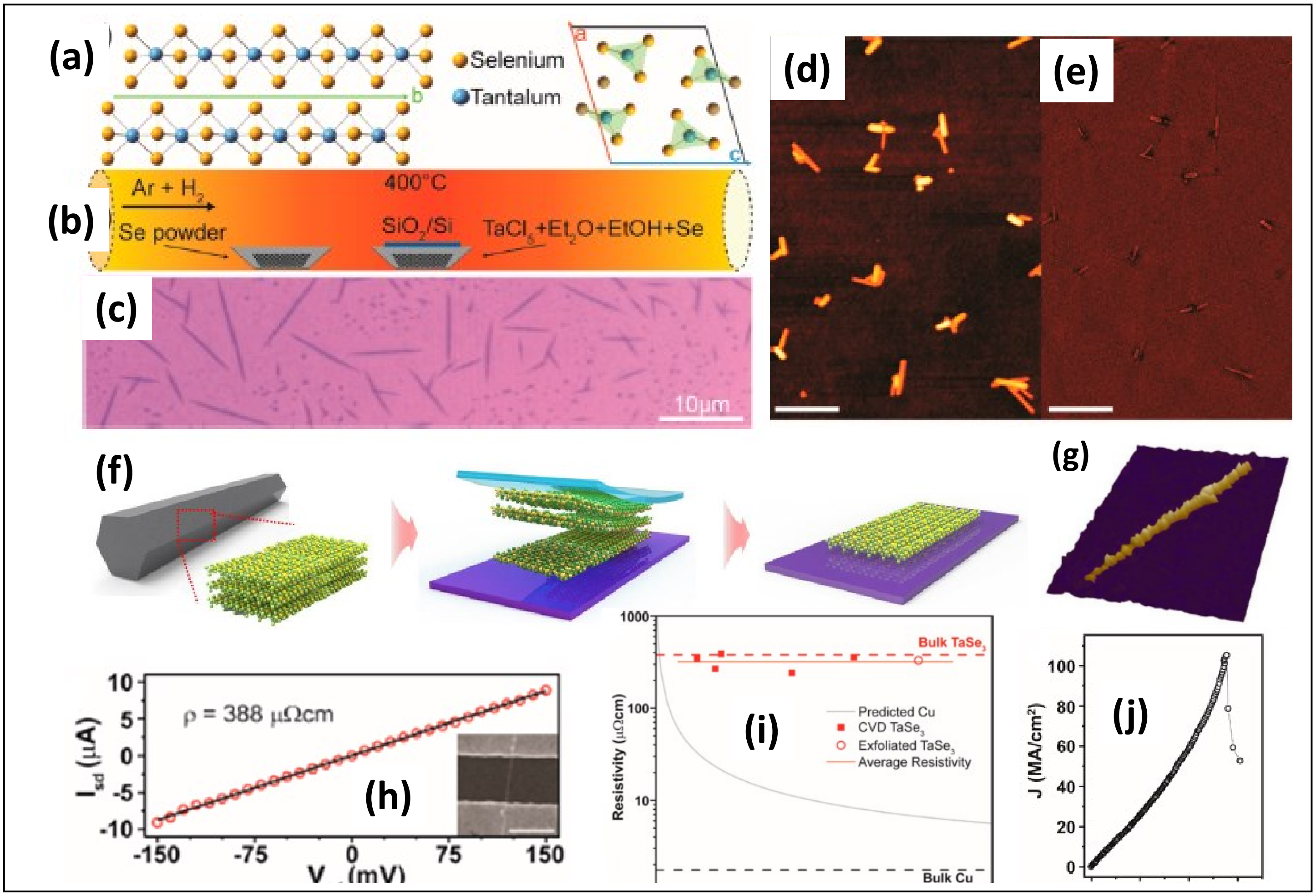

Figure 9: (a) Crystallographic structure of $TaSe_3$, (b) schematic diagram of the CVD set up for the growth of $TaSe_3$ nanowires, (c) optical image and (d, e) SEM image of a population of $TaSe_3$ nanowires, Reprinted with permission from Ref. no-78, copyright (2019) American chemical Society (f) schematic illustration of mechanical exfoliation of $TaSe_3$ flakes from bulk $TaSe_3$, (g) 3D monolayer of a quasi-1D $TaSe_3$ nanoribbon on SiO_2/Si substrate, Reproduced with permission from ref. no.-107, copyright (2019) Multidisciplinary Digital Publishing Institute, (h) source-drain current vs. voltage for a 11.6 nm $TaSe_3$ nanowire in a 2 electrode configuration with inset showing the SEM image of the device, (i) resistivity as a function of bundle width of CVD grown $TaSe_3$ nanowire and comparison with bulk Cu and exfoliated $TaSe_3$, (j) current density response of a 7x7 nm^2 nanowire as a function of V_{ds} with a failure at current density of 108 A/cm^2, , Reprinted with permission from Ref. no-78, copyright (2019) American chemical Society.

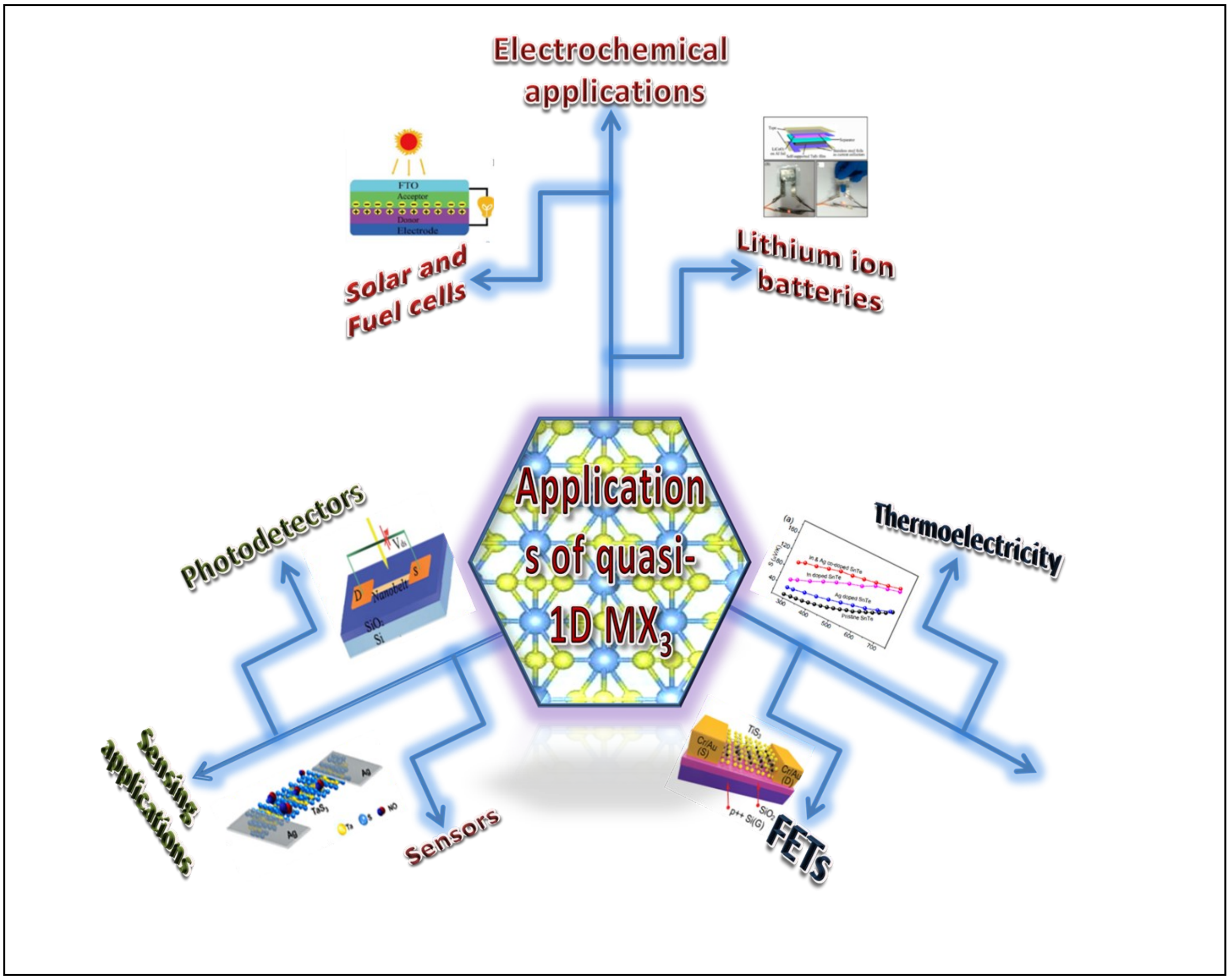

Figure 10: Schematic diagram showing various applications of MX_3, solar and fuel cell fig.- Republished with permission from ref. no.-33; permission conveyed through Copyright Clearance Center, Inc, lithium ion batteries fig.- Reprinted with permission from Ref. no-134, copyright 2015 American chemical Society, sensors fig.- Reprinted with permission from Ref. no-133, copyright (2018) American Chemical Society, photodetectors fig.- Republished with permission from ref. no.-129; permission conveyed through Copyright Clearance Center, Inc, FETs fig.-Reproduced with permission from ref. no.-124, copyright (2015) Royal Society of Chemistry, thermoelectricity fig.- Republished with permission from ref. no.-157; permission conveyed through Copyright Clearance Center, Inc, central str. Fig.- reproduced with permission from Ref- (6), copyright (2015) Springer Nature

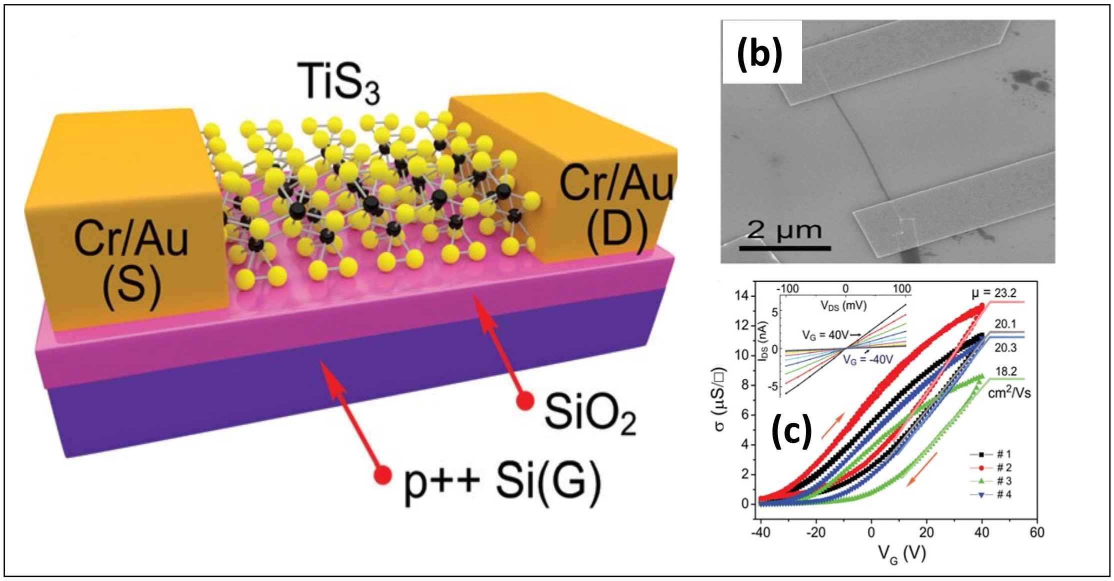

Figure 11: Few layered TiS_3 FETs (a) schematic and (b) SEM image of a typical FET, (c) Conductivity vs. gate voltage dependencies for four different fabricated FETs with the inset showing the drain source current vs. drain source voltage dependencies at different gate voltages. Electrical characterization of TiS_3 nanosheets, Reproduced with permission from ref. no.-124, copyright (2015) Royal Society of Chemistry.

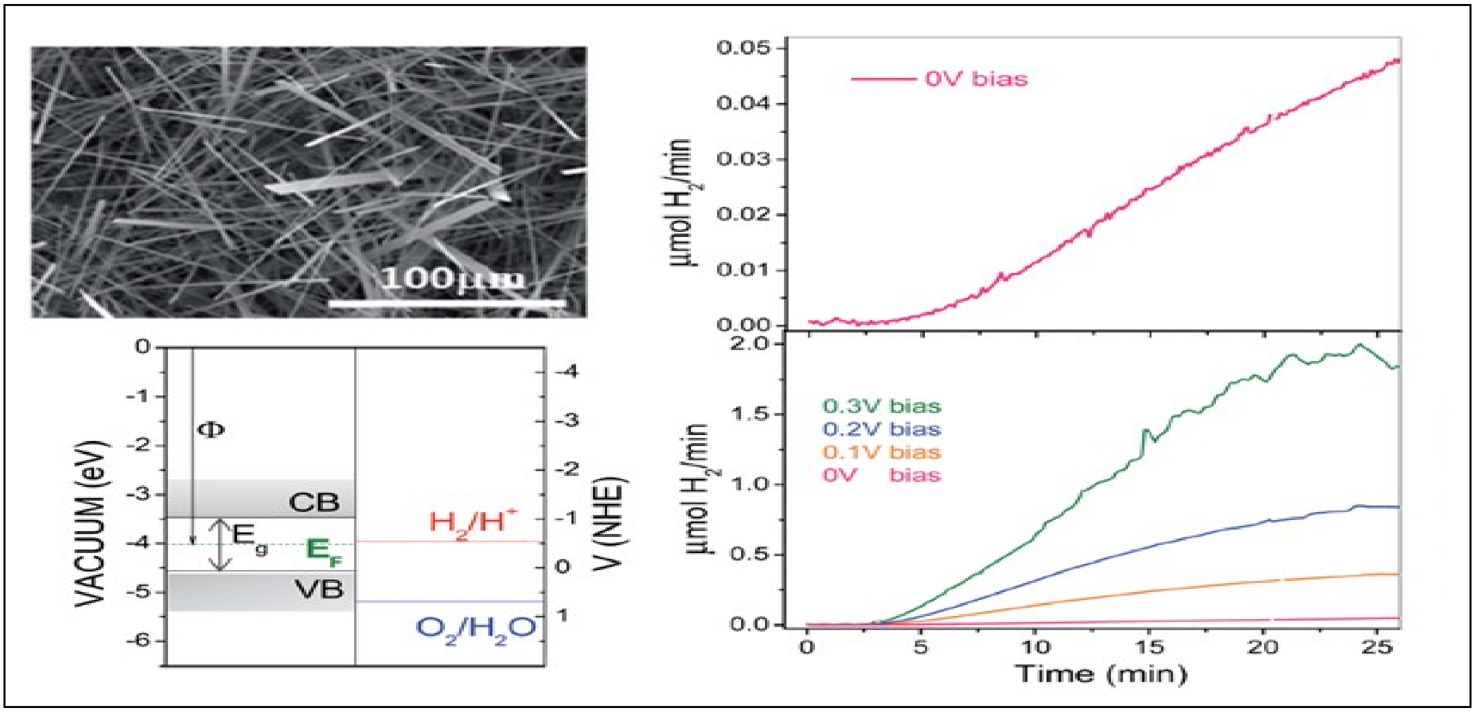

Figure 12: Hydrogen photogeneration properties of MX_3 nanostructures: (a) SEM image, (b) conduction and valence band energy levels in potentials (V vs. NHE) and energy (eV vs. vacuum) scales with redox potentials for the water-splitting half reactions at pH =9.0 vs NHE of TiS_3 nanoribbons. Hydrogen evolution flow of TiS_3 nanoribbons at (c) 0.0 V (d) at different bias potentials, Republished with permission from ref. no.-144; permission conveyed through Copyright Clearance Center, Inc.

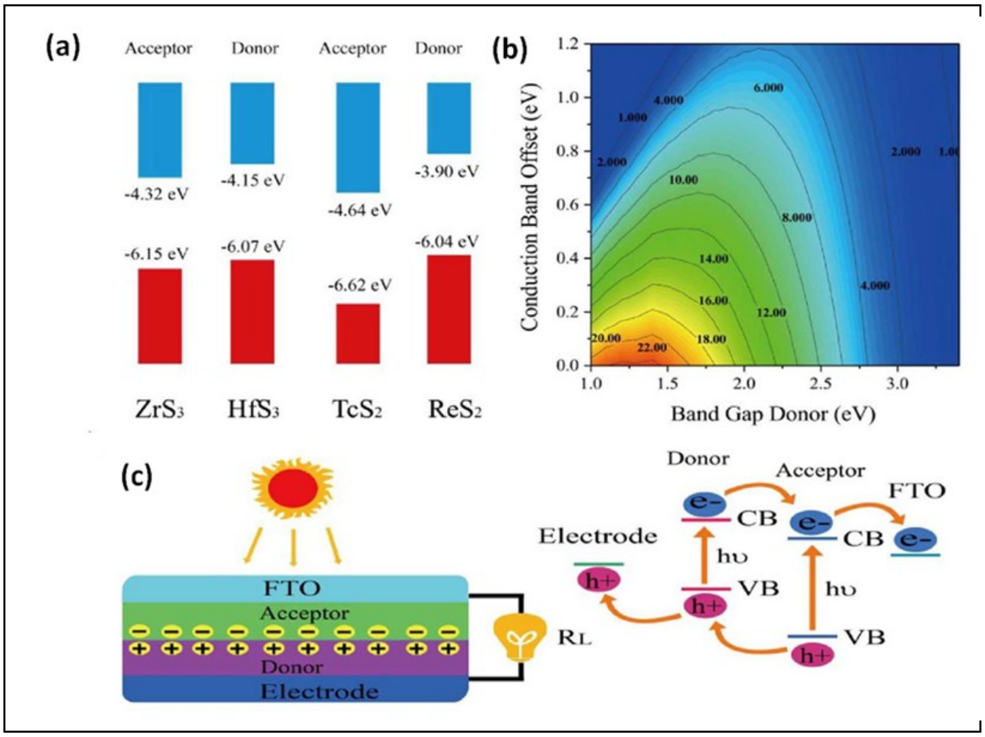

Figure 13: Application of MX_3 heterostructures for solar cells: (a) Band offsets of ZrS_3/HfS_3 and TcS_2/ReS_2 with the vacuum level as zero reference, (b) PCE contour obtained as a function of donor band gap and conduction band offset, (c) schematic illustration of thin film solar cells with mechanism, Republished with permission from ref. no.-33; permission conveyed through Copyright Clearance Center, Inc.

.

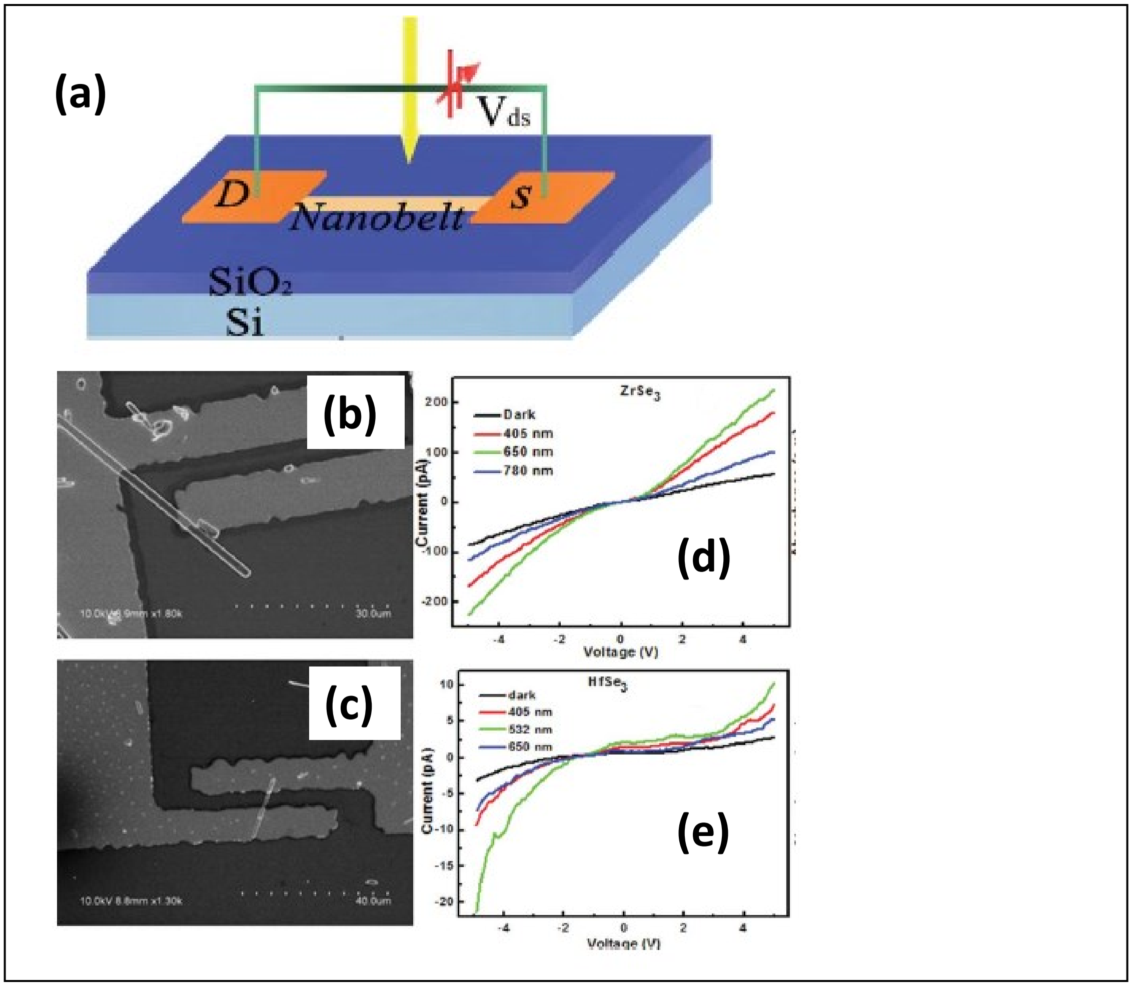

Figure 14: (a) schematic illustration of the $ZrSe_3$ and $HfSe_3$ single nanobelt photodetector, SEM images of (b) $ZrSe_3$, (c) $HfSe_3$ photodetector, I-V characteristics of the (d) $ZrSe_3$, (e) $HfSe_3$ nanobelt photodetector, Republished with permission from ref. no.-129; permission conveyed through Copyright Clearance Center, Inc.

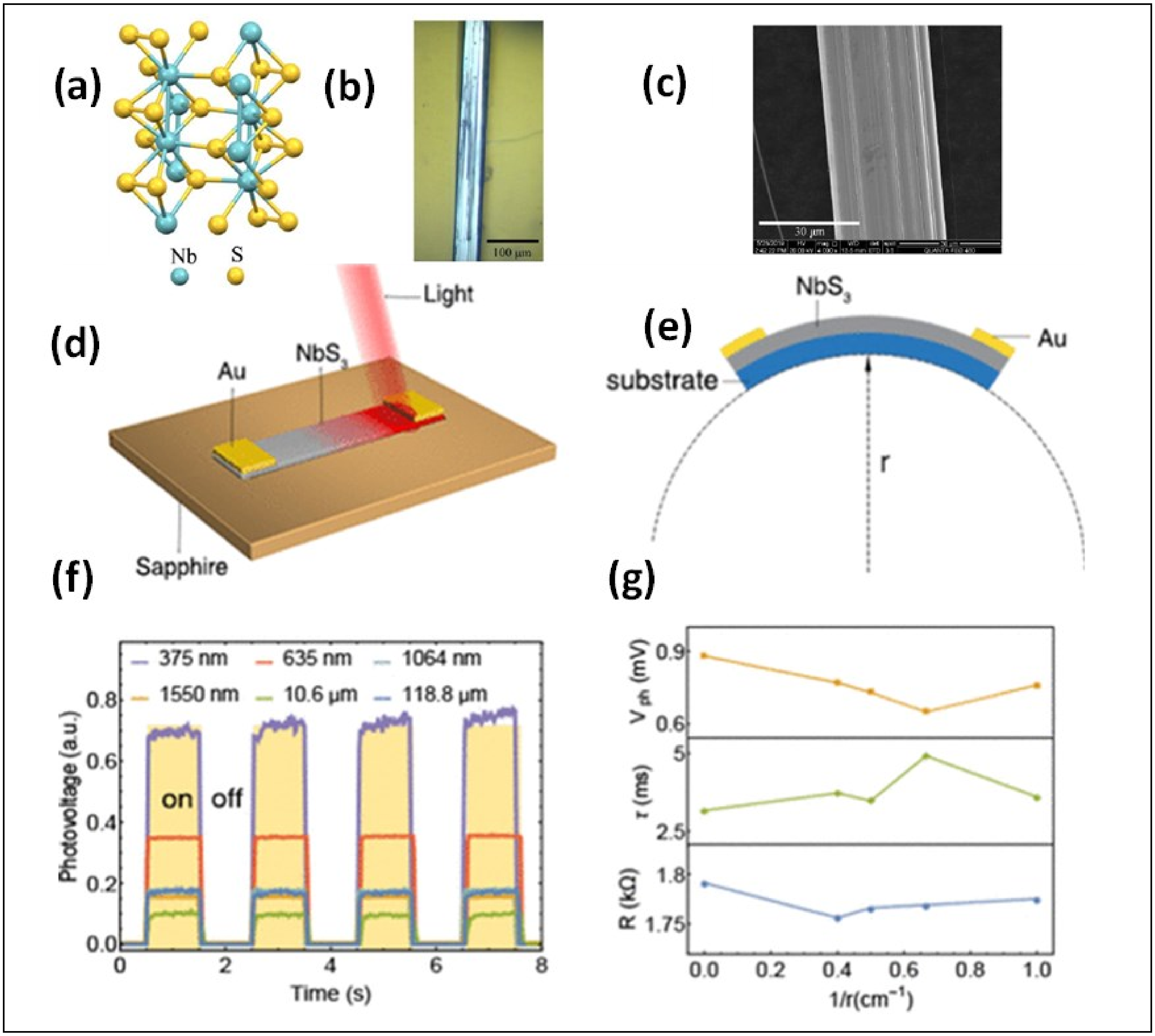

Figure 15: (a) Crystal structure of NbS_3, (b) Optical microscopic and (c) SEM image of NbS_3 crystal. (d) schematic diagram of a (d) NbS_3 photodetector and (e) bending condition, (f) on-off curves of photovoltage at room temperature and (g) resistance, response time and photovoltage under different bending condition, Reprinted with permission from Ref. no-151, copyright (2020) American Chemical Society

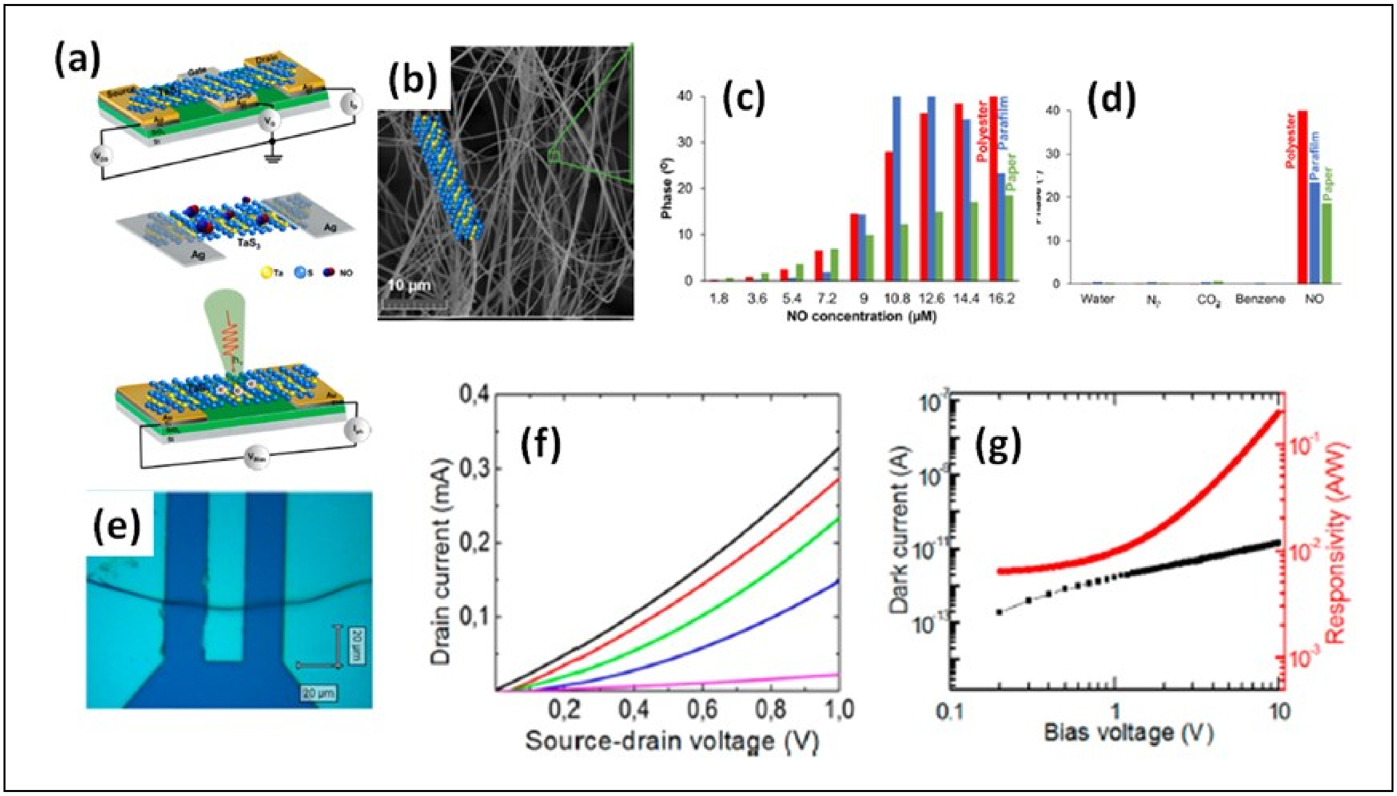

Figure 16: (a) Schematic representation of the FET, NO gas sensor and photodetector based on TaS_3 nanofiber, (b) SEM image of the TaS_3 nanofibers, (c) impedance phase response of the gas sensor fabricated on different substrates (polyster, parafilm, paper) as functions of NO concentrations, (d) selectivity studies of the sensors fabricated on different substrates, (e) optical image of a FET based on TaS_3 fiber, (f) FET transistor characteristics curves at different gate voltages (violet: -1 V, blue: 0 V, green: 1V, red: 2V, black: 3V), (g) dark (black) and its responsivity (red) curves, Reprinted with permission from Ref. no-133, copyright (2018) American Chemical Society

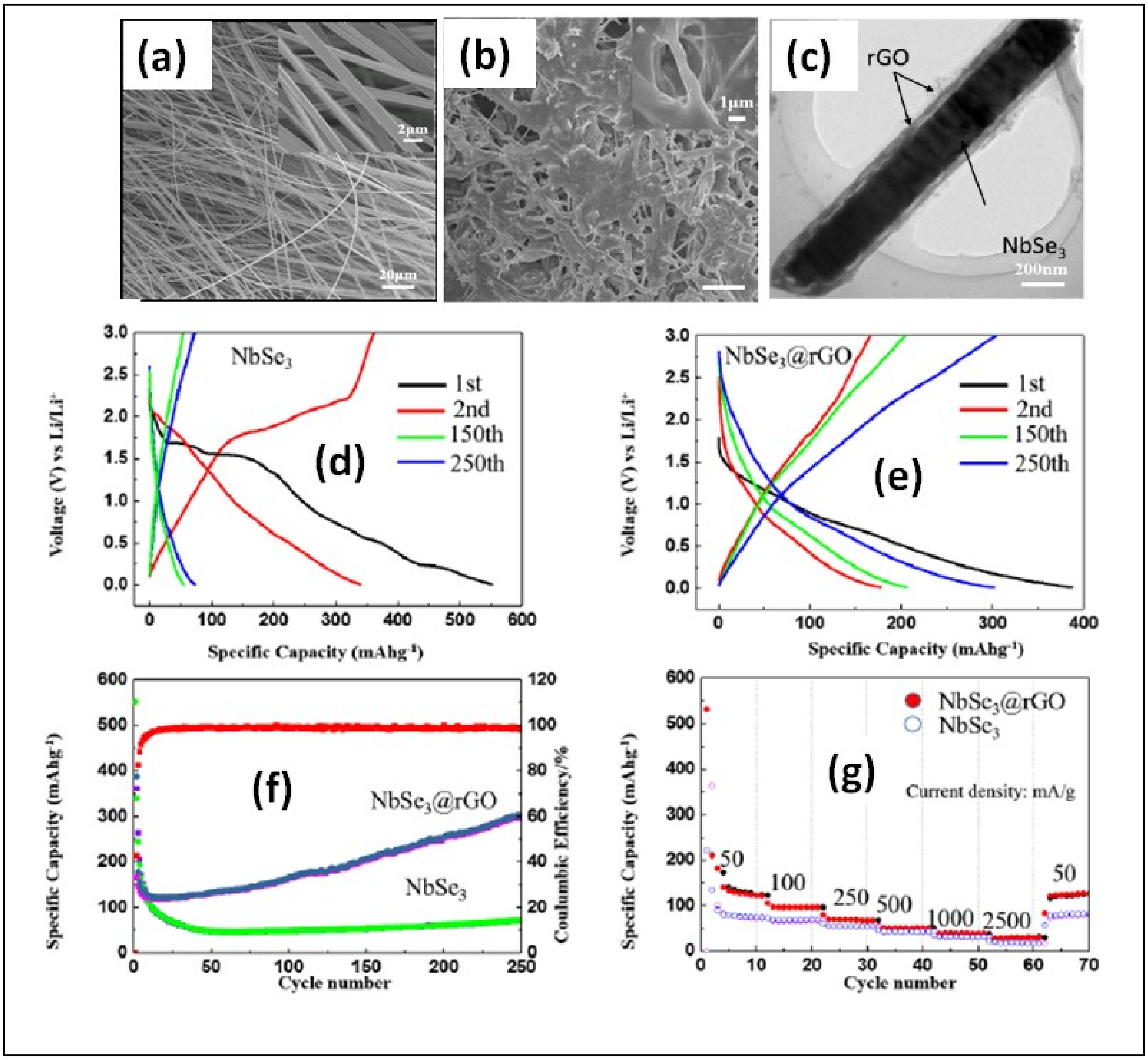

Figure 17: SEM images of (a) $NbSe_3$ and (b) $NbSe_3$@rGO, (c) TEM image of $NbSe_3$@rGO. Electrochemical performance of $NbSe_3$ and $NbSe_3$@rGO in the voltage range of 0.005-3 V vs. Li/Li^+. Discharge and charge curves of (d) $NbSe_3$ and (e) nanobelts. (f) Cycling performance of $NbSe_3$@rGO and $NbSe_3$ nanobelts at a current density of 100 mA/g. (g) Rate performance of $NbSe_3$@rGO and $NbSe_3$ nanobelts, Reprinted from ref. no-155, Copyright (2017) with permission from Elsevier.

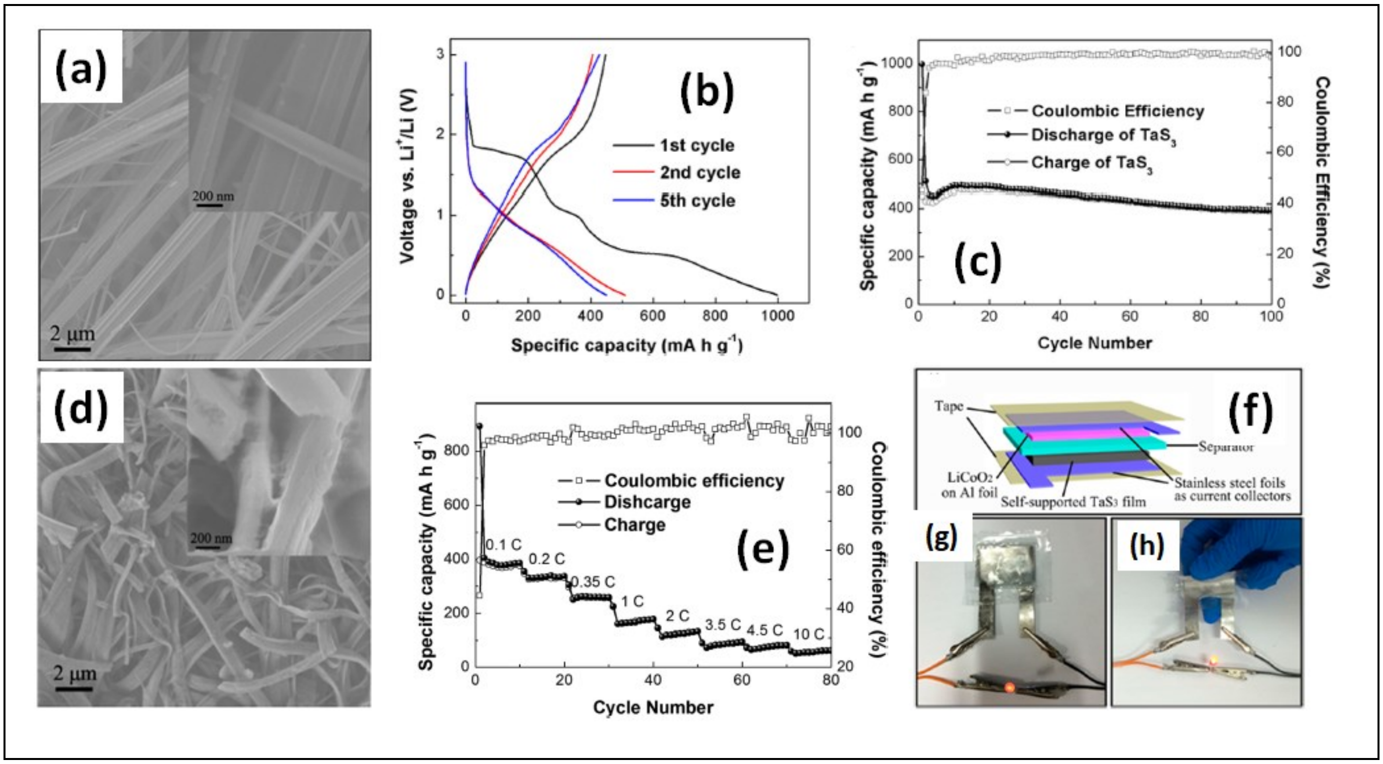

Figure 18: (a) SEM image of TaS_3 nanowires, (b) voltage profiles of lithium ion battery based on TaS_3 nanowires cycled between 0.001 and 3 V vs. Li+/Li at a cycling rate of 0.1C, (c) Capacity and columbic efficiency-cycle number curves of TaS_3 nanowire curves of TaS_3 nanowire electrode at a cycling rate of 0.1C, (d) SEM image of the nanowires after long cycling tests, (e) rate performance and columbic efficiency-cycle number curves of TaS_3 electrodes as a function of discharge rate (0.1-10C) (f-h) schematic of the fabricated flexible lithium ion battery with photographs showing glowing of LED under flat and bent states, Reprinted with permission from Ref. no-134, copyright (2015) American chemical Society
